# CellBinDB: a large-scale multimodal annotated dataset for cell segmentation with benchmarking of universal models

**DOI:** 10.1093/gigascience/giaf069

**Published:** 2025-06-24

**Authors:** Can Shi, Jinghong Fan, Zhonghan Deng, Huanlin Liu, Qiang Kang, Yumei Li, Jing Guo, Jingwen Wang, Jinjiang Gong, Sha Liao, Ao Chen, Ying Zhang, Mei Li

**Affiliations:** BGI Research, Shenzhen 518083, China; School of Artificial Intelligence, University of China Academy of Science, Beijing 100049, China; BGI Research, Shenzhen 518083, China; School of Artificial Intelligence, University of China Academy of Science, Beijing 100049, China; BGI Research, Shenzhen 518083, China; BGI Research, Shenzhen 518083, China; BGI Research, Shenzhen 518083, China; BGI Research, Shenzhen 518083, China; BGI Research, Shenzhen 518083, China; BGI Research, Shenzhen 518083, China; BGI Research, Shenzhen 518083, China; BGI Research, Shenzhen 518083, China; BGI Research, Shenzhen 518083, China; BGI Research, Shenzhen 518083, China; BGI Research, Shenzhen 518083, China

**Keywords:** dataset, cell segmentation, benchmark, universal models

## Abstract

In recent years, cell segmentation techniques have played a critical role in the analysis of biological images, especially for quantitative studies. Deep learning–based cell segmentation models have demonstrated remarkable performance in segmenting cell and nucleus boundaries, but they are typically tailored to specific modalities or require manual tuning of hyperparameters, limiting their generalizability to unseen data. Comprehensive datasets that support both the training of universal models and the evaluation of various segmentation techniques are essential for overcoming these limitations and promoting the development of more versatile cell segmentation solutions. Here, we present CellBinDB, a large-scale multimodal annotated dataset established for these purposes. CellBinDB contains more than 1,000 annotated images, each labeled to identify the boundaries of cells or nuclei, including 4′,6-diamidino-2-phenylindole, single-stranded DNA, hematoxylin and eosin, and multiplex immunofluorescence staining, covering over 30 normal and diseased tissue types from human and mouse samples. Based on CellBinDB, we benchmarked 8 state-of-the-art and widely used cell segmentation technologies/methods, and our further analysis reveals that complex cell shapes reduce segmentation accuracy while higher image gradients improve boundary detection, offering insights for refining segmentation strategies across diverse imaging scenarios.

## Introduction

Cell/nuclear staining technology enhances the visualization of cell or subcellular resolution level in microscope images, thereby contributing to quantitative analysis in biomedical research [1]. As microscopy advances to study complex biological structures in great detail and produce rich, dense images [2], there is an urgent need for automated methods to extract cellular information by accurately segmenting cells and nuclei [1, 3], especially important in spatial transcriptomics. Despite the significant progress made by deep learning methods in segmenting cell and nuclear boundaries in limited latent features of some specific images [4–14], challenges remain in developing universally applicable models. One of the key obstacles is the lack of large and diverse annotated image datasets that can support the training and evaluation of robust universal models [15–18].

Previous works have made significant contributions by releasing several public datasets for training and evaluating deep learning models. Although all important advances, they are limited in their ability to simultaneously meet the requirements for large-scale, multiple staining techniques and diverse tissue types, and thus they are unable to fulfill the needs for universal models. Table 1 shows the annotated stained datasets that have been actively used by the research community in recent years. Some datasets are limited in scale or tissue-type richness, such as MoNuSeg [15], IEEE_TMI_2019 [19], and the fluorescence image dataset published by Kromp et al. in 2020 [20], all of which contain fewer than 100 images. A slightly larger dataset, Lizard [21], consists of only 1 tissue type. Table 1 also shows that most of the mentioned datasets are based on hematoxylin and eosin (H&E) staining, which is the most common type of staining in routine pathology [22], followed by immunofluorescence (IF) staining, which is more frequently employed in research settings. The Cellpose dataset contains a large number of nonmicroscope images of repeated objects, such as fruits, vegetables, and rocks, and microscope images of bacteria. When considering mammalian stained cell images, a total of 613 images were included in the comparative analysis. NuInsSeg [22] is limited to a single staining type of H&E. Similarly, BBBC039 [23] is limited to Hoechst, a membrane-permeable fluorescent dye. Additionally, there are also 3 large-scale and more diverse datasets. The Data Science Bowl 2018 featured a dataset (BBBC038v1) [18] with 37,333 manually annotated cell nuclei, but datasets commonly utilized for training and benchmarking segmentation models in stained cell images are still significantly larger by comparison. TissueNet [7] emphasizes immunostaining while lacking histological staining and nucleic acid staining. The NeurIPS 2022 dataset [24] aims to achieve richness in image modalities, with constrained quantity in each image type, especially in the case of stained images.

**Table 1:** Publicly available annotated cell segmentation datasets

Dataset	Staining type	Image tiles	Cell number	Organs	Tile size (pixels)
MoNuSeg [15]	H&E	44	28,846	9	1,000 × 1,000
IEEE_TMI_2019 [19]	H&E	80	25,645	8	512 × 512 and 1,000 × 1,000
Kromp et al. [20]	IF/DAPI	79	7,813	5	550 × 430 to 1,360 × 1,024
Lizard [21]	H&E	238	495,179	1	512 × 512
Cellpose [5]	IF	613	About 70,000	Unknown	147 × 164 to 2,560 × 2,160
NuInsSeg [22]	H&E	665	30,698	31	512 × 512
BBBC038v1 [18]	DAPI/Hoechst/H&E	841	37,333	Unknown	256 × 256
BBBC039 [23]	Hoechst	200	23,165	Unknown	520 × 696
TissueNet [7]	IF	3,200	1.3 million	9	512 × 512
NeurIPS 2022 cell segmentation competition dataset [24]	Jenner—Giemsa/IF	713	134,710	Unknown	512 × 512 to 4,096 × 4,096
**CellBinDB**	**DAPI/ssDNA/H&E/mIF**	**1/044**	**109,083**	**35**	**256 × 256 and 512 × 512**

Recently, many efforts have focused on developing universal methods and demonstrating robust performance on unseen datasets [5–7, 9, 25, 26]. SAM (RRID:SCR_023680) [25], a method based on the self-attention mechanism, has the capability of zero-shot generalization of new image distributions and tasks. Cellpose1 (RRID:SCR_021716) [5] is based on the U-Net architecture, which can precisely segment cells from diverse image types without model retraining or parameter adjustments. Cellpose3(RRID:SCR_021716) [6], an improved version, specializes in out-of-box segmentation of noisy, blurred, or undersampled images. DeepCell(RRID:SCR_022197) [7], a deep learning algorithm for accurate whole-cell segmentation, achieves human-level segmentation performance by combining a ResNet50 backbone network and a feature pyramid network. MEDIAR [26] emerged as the state of the art (SOTA) in the NeurIPS 2022 multimodal cell segmentation competition. StarDist [9] utilizes star-convex polygons to represent cell shapes, enabling accurate cell localization even under challenging conditions, especially when dealing with overlapping cells. However, the diversity of currently available models and the inconsistency of segmentation quality metrics make it difficult to evaluate their relative performance based on literature descriptions [27]. Therefore, it is necessary to evaluate these universal algorithms on a new unseen annotated dataset.

In this study, we present a new large-scale, multimodal annotated dataset, CellBinDB, containing images of 4 staining types (4′,6-diamidino-2-phenylindole [DAPI], H&E, single-stranded DNA [ssDNA], and multiplex immunofluorescence [mIF]) derived from over 30 human and mouse tissues. The primary statistic of CellBinDB is presented in the last row of Table 1. Unlike previous efforts that may be limited in tissue type coverage [23, 24], CellBinDB includes a wide range of both normal and diseased tissues, making it one of the most comprehensive tissue-type datasets available. The images included in the dataset were obtained from the 10x Genomics platform, as well as from new experiments based on Stereo-seq technology. Given that manual annotation is a time-intensive task, it is significantly limiting the scale of datasets. Besides, model annotation methods are affected by model style and cannot guarantee accuracy. To balance quality and efficiency in annotating this multimodal dataset, we used a combination of manual and semiautomatic annotation strategies. To eliminate potential biases in the reference model [25], the model segmentation results were manually revised by a trained team of professional annotators and then checked by experts, ensuring that all annotations passed through 2 rounds of review. We made CellBinDB available to the research community and evaluated the performance of some general models on the dataset, providing recommendations for the selection of cell segmentation models in different scenarios. Furthermore, several cell segmentation models were fine-tuned using CellBinDB and subsequently evaluated on the independent IEEE_TMI_2019 and Lizard dataset. The fine-tuned models showed significant performance improvements (Supplementary Fig. S1), and these results substantiate the utility of CellBinDB as a valuable resource for advancing model development and performance optimization in cell segmentation research.

## Results

### Dataset

**Figure 1:**
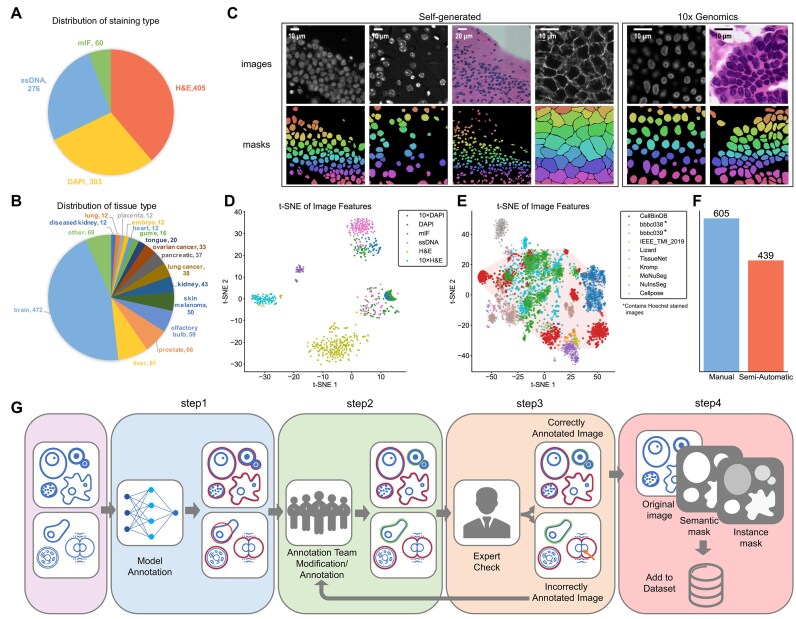
CellBinDB overview. (A) Distribution of staining types in CellBinDB. (B) Distribution of tissue types in CellBinDB, where tissue types with fewer than 10 samples are included in "other"; for details, see Supplementary Table S1. (C) Examples of CellBinDB images with scale bar and instance ground-truth annotations, from left to right: column 1, ssDNA; column 2, DAPI; column 3, H&E; column 4, mIF; column 5, 10x Genomics DAPI; column 6, 10x Genomics H&E. The first row provides the original microscope images, and the second contains the instance annotation masks. (D) Scatterplot of t-SNE demonstrates the diverse spread of data by different staining types and sources. (E) Scatterplot of t-SNE demonstrates the diversity of CellBinDB compared to previous datasets. (F) The number of manual and semiautomatic annotations in CellBinDB. (G) The dataset annotation process includes 4 steps: 1, model annotation; 2, annotation team modification/reannotation (depends on the model annotation results); 3, expert review, go to the next step if the annotations are correct, otherwise return to the second step for modification; and 4, add the original image and the 2 masks to the dataset.

In this study, we propose CellBinDB, a dataset comprising 1,044 annotated microscopy images and 109,083 cell annotations. This dataset contains 4 staining types: DAPI, ssDNA, H&E, and mIF (Fig. 1A). CellBinDB encompasses samples derived from human and mouse species, covering over 30 histologically diverse tissue types, including disease-relevant tissues (Fig. 1B). The images in CellBinDB come from 2 sources: 844 mouse images were from in-house experiments based on Stereo-seq technology and 200 human images obtained from the open-access platform 10x Genomics. We annotate all images in CellBinDB and offer 2 types of image annotations: semantic and instance masks (Fig. 1C). Annotation is a combination of manual and semiautomatic methods, with 60% manual annotation and the rest from semiautomatic annotation (Fig. 1F). The annotation workflow is shown in Fig. 1G, with all annotations double-checked by experts to ensure quality. Additional details are in the Methods section. CellBinDB has diverse features that make it suitable for training generalized segmentation models. To visualize the image features of CellBinDB, we applied t-distributed stochastic neighbor embedding (t-SNE [28, 29]) to neural network–learned image features, revealing clusters that generally align by staining type (Fig. 1D). The ssDNA and DAPI staining types show similar features, while there are significant differences between images of the same staining type from the 2 sources. CellBinDB demonstrates a broader feature distribution compared to existing datasets with overlapping staining types, encompassing characteristics from most major public benchmarks (Fig. 1E). However, certain staining modalities (Hoechst stain in BBBC038/039) remain outside its current scope. Supplementary Table S1 provides further details about CellBinDB.

### Benchmark performance

We evaluated several widely recognized segmentation models on CellBinDB, including specialized cell segmentation models: Cellpose1 [5], Cellpose3 [6], StarDist [9], DeepCell [7], MEDIAR [24, 26], a model for general segmentation (SAM [25]), and a software widely used in biomedicine and life sciences: CellProfiler(RRID:SCR_007358SAM) [8]. Among them, Cellpose1, Cellpose3, StarDist, and DeepCell are models based on U-Net; MEDIAR and SAM are models based on Transformer; and CellProfiler is a model based on machine learning. Since the models were trained on different training sets, a fair evaluation was conducted using CellBinDB, a dataset that none of the models had been trained on. The evaluation on CellBinDB was first performed on the entire dataset, followed by separate evaluations on each staining type. In the H&E subset evaluation, we also included HoverNet [30], a specialized model specifically designed for pathological H&E image analysis, in our comparative assessment.

#### Evaluation results on the entire dataset

**Figure 2:**
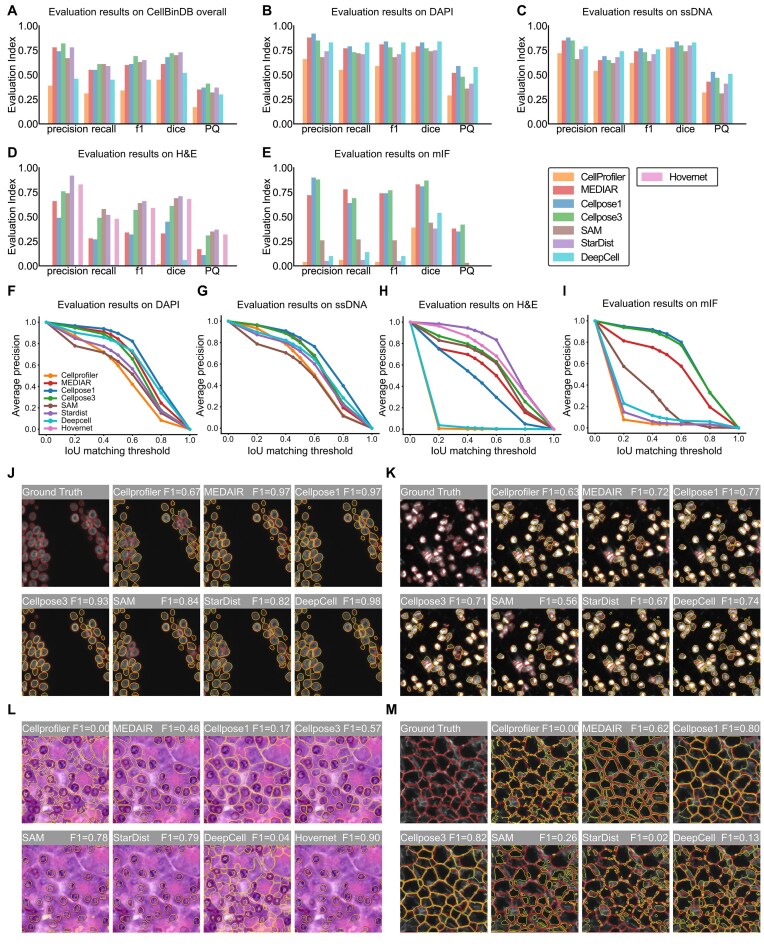
Evaluation of model performance on the entire dataset and by staining type. (A) Evaluation of model performance on the entire dataset, with bar charts of precision, recall, F1 score, Dice, and PQ. (B) Model performance evaluation results on DAPI-stained images. (C) Model performance evaluation results on ssDNA-stained images. (D) Model performance evaluation results on H&E-stained images. (E) Model performance evaluation results on mIF-stained images. (F–I) AP-IoU curves of the segmentation results of the model on 4 stains (DAPI, ssDNA, H&E, mIF). The IoU threshold quantifies the match between the predicted mask and the ground-truth mask. (J–M) Examples of segmentation results for each model on 4 stains (DAPI, ssDNA, H&E, mIF). The ground truth is represented by the red line, while the model prediction is represented by the yellow line.

The initial objective was to assess the capacity of each model to perform segmentation on multimodal cell images (Fig. 2A). The experimental results demonstrate that most of them exhibited excellent performance, except for CellProfiler and DeepCell. Of the models evaluated, Cellpose3 demonstrated the most optimal performance and was the most highly recommended (precision: 0.82, recall: 0.61, F1 score: 0.70, Dice: 0.72). In contrast, the performance of Cellpose1, with a similar architectural design, was less impressive in terms of a lower F1 score than Cellpose3. It is hypothesized that this inferior performance is attributable to the smaller and less diverse training dataset employed for Cellpose1 in comparison to Cellpose3. Similarly, DeepCell demonstrated suboptimal overall performance due to the limited diversity and lack of variability in its training dataset, which consisted exclusively of fluorescent staining images. Furthermore, the machine learning–based CellProfiler model demonstrated the poorest performance among the evaluated models. In addition, compared with the F1 score, all models obtained lower PQ indexes, indicating that the models did not capture complex or fuzzy boundaries well and were not able to correctly separate closely adjacent instances, resulting in overlapping or sticky boundaries. Future model development could focus on improving the boundary accuracy of instance segmentation, enhancing the detection recall rate, and refining classification accuracy to further optimize model performance.

#### Evaluation on 4 staining types

We next evaluated the segmentation capabilities of each model on 4 staining types. Figure 1 illustrates the diverse characteristics of images in CellBinDB. The evaluation of the models on the 4 staining types highlights the varying levels of difficulty across the stains and the strengths of each model. In detail, most models scored higher on DAPI-stained images (Fig. 2B, F, J) and ssDNA-stained images (Fig. 2C, G, K). Notably, Cellpose1, DeepCell, and MEDIAR performed exceptionally well on these 2 stain types and are highly recommended. In contrast, significant variability was observed in model performance on H&E-stained images (Fig. 2D, H, L) and mIF-stained images (Fig. 2E, I, M). On H&E-stained images, StarDist, SAM, HoverNet, and Cellpose3 performed well, whereas other models showed poor results or even failed. We attributed StarDist’s superior performance to its specialized weights trained for H&E-stained images and the effective segmentation of HoverNet to its design specifically for H&E-stained pathology images, while SAM and Cellpose3 benefited from their large training datasets, enabling them to effectively segment H&E images. The poor performance of CellProfiler and DeepCell likely stemmed from their incorrect interpretation of the foreground and background (Fig. 2L). mIF is a membrane stain that has the opposite color characteristics compared to DAPI and ssDNA, showing that the inside of the cell is dark. On mIF-stained images, Cellpose1, Cellpose3, and MEDIAR are the most recommended. While SAM was able to process mIF images, its performance was relatively poor. The remaining models failed to effectively perform cell segmentation on mIF images because they mistakenly segmented the bright cell membranes as cells. Overall, H&E-stained and mIF-stained images pose greater segmentation challenges than other stain types.

On CellBinDB, we also matched the model’s predictions to the ground-truth masks at different matching accuracy thresholds based on the standard intersection-over-union (IoU) metric. We evaluated performance using the average precision (AP) metric. The abovementioned top-performing models maintained good performance at the commonly used IoU threshold of 0.5 and higher IoUs, which benchmark the ability to accurately segment cell boundaries.

**Figure 3:**
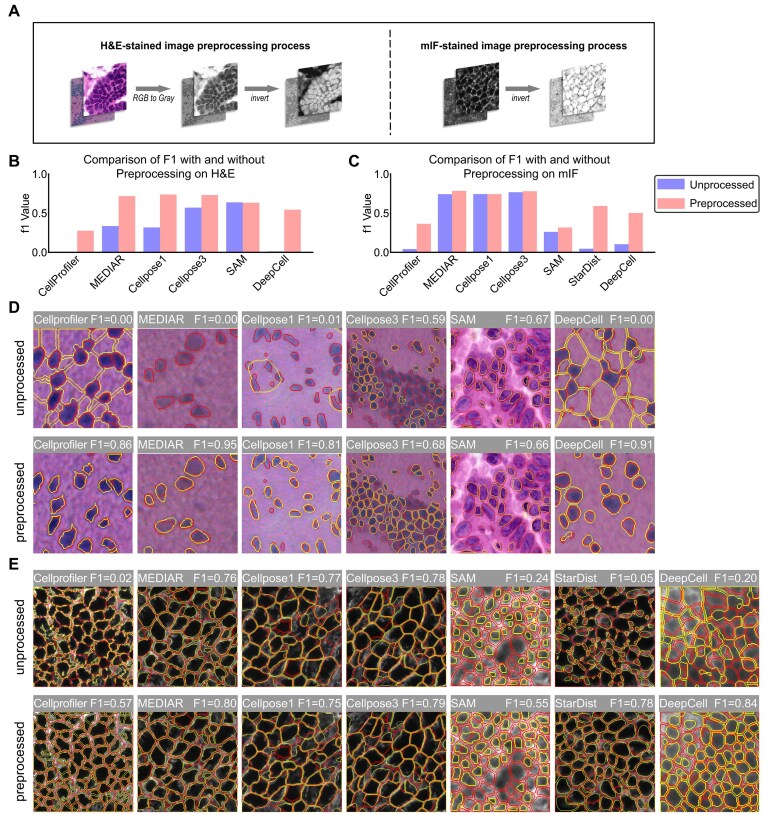
Model performance after adding preprocessing. (A) Preprocessing of H&E- and mIF-stained images. H&E-stained images include 2 steps: grayscale conversion and color inversion, while mIF-stained images only require color inversion. (B) Comparison of F1 scores before and after adding preprocessing for the model with improved performance on H&E-stained images. (C) Comparison of F1 scores before and after adding preprocessing for the model with improved performance on mIF-stained images. (D) Example of segmentation results before and after adding preprocessing on H&E-stained images. (E) Example of segmentation results before and after image preprocessing on mIF-stained images.

Considering the specificity of H&E-stained images, some models perform additional preprocessing of the image, such as converting RGB to grayscale or processing color deconvolution [31], to make the image features more consistent with the fluorescent stained image. With the exception of StarDist and HoVerNet—both specifically optimized for H&E image analysis—all 6 comparative models underwent standardized preprocessing involving grayscale conversion and color inversion (Fig. 3A). Quantitative evaluation revealed that preprocessing substantially enhanced segmentation accuracy for the initially underperforming models, while models with baseline high performance exhibited marginal improvements or maintained their original accuracy levels (Fig. 3B, D). Among them, DeepCell exhibited the most significant performance improvement; without preprocessing, it could hardly segment H&E images. After adding preprocessing, CellProfiler and DeepCell were able to correctly interpret the foreground and background, and the performance of MEDIAR and Cellpose1 also improved to varying degrees. These results indicate that models originally designed for fluorescent images could be more effectively adapted to RGB image segmentation through preprocessing. After completing the above preprocessing, we recommended using Cellpose1, Cellpose3, or MEDIAR for cell segmentation of H&E-stained images. Similarly, this approach can be extended to mIF staining, with several previously underperforming models benefiting, particularly StarDist (Fig. 3C, E), but it still did not outperform Cellpose1, Cellpose3, and MEDIAR. The preprocessing process is shown in Fig. 3A. Moreover, SAM demonstrates the capacity to process a range of stain types with consistent efficacy, although its performance is not as robust as that of models tailored for cell segmentation. This is believed to be due to the training data used for SAM, which are likely to contain a significant number of images that are not cells. In the absence of predefined settings or preprocessing, it is recommended to use SAM as a model capable of multimodal cell image segmentation.

### Factors affecting cell segmentation performance

Next, we analyzed the factors that affect the model’s ability to segment cells from both biological and non-biological perspectives.

#### Evaluation of impact of cell morphology

**Figure 4:**
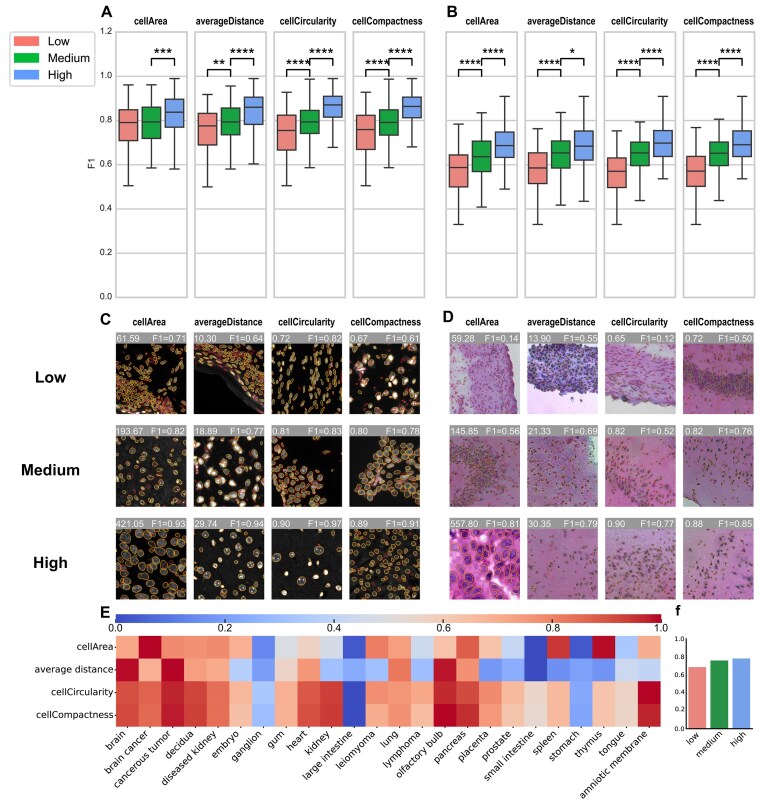
Evaluation of the impact of cell morphology. (A) For fluorescent-stained images (DAPI and ssDNA, Cellpose1 as an example), 4 indicators (cellArea, averageDistance, cellCircularity, cellCompactness) are used to evaluate the impact of cell morphology on segmentation performance. The vertical axis of the box plot is the F1 score, and the horizontal axis is the sample divided into 3 parts: low, medium, and high according to the tertile of each indicator. The significance mark line and *P* value are added to the box plot. The number of “*” from 1 to 4 represents a *P* value less than 0.05, 0.01, 0.001, and 0.0001. (B) Same as panel A, with results on H&E-stained images. (C) Fluorescent-stained images display low, medium, and high instances under 4 indicators. (D) H&E-stained images display low, medium, and high instances under 4 indicators. (E) Display of normalized scores for the above 4 metrics by tissue type. (F) Based on the average of the four cell morphology evaluation indicators for each image, the cells are categorized into 3 groups: low, medium, and high. The bar graph shows the relationship with the F1 score.

We evaluated a series of cell morphology metrics, including the cell morphology evaluation metrics in BIDCell [32], as well as an original metric, cell average distance, for evaluating cell density. The 4 metrics that exhibited the strongest correlation with the F1 score in the case of CellBinDB were cell area, average distance, cell circularity and cell compactness. The results of the experiment indicate that an increase in the metrics is associated with an improvement in the performance of the segmentation. To illustrate, Cellpose1 demonstrated the most favorable performance in fluorescence staining (Fig. 4A, C), whereas StarDist exhibited the most optimal results in H&E-stained images (Fig. 4B, D). The full set of conclusions, which includes all models, can be found in the Supplementary Fig. S2. The fluorescence staining images do not exhibit a notable distinction between the low and medium categories with respect to the indicator cellArea. This observation may be attributed to the intrinsic characteristics of the data, with the cell areas of most images displaying cell areas concentrated within the region with lower values.

Cell morphology is directly related to tissue type. We classify the images in the dataset according to tissue type, calculate the above 4 indicators, and then normalize them. These results are presented in Fig. 4E, which provides an overview of the difficulty level of cell segmentation for different tissue types and indicates the potential challenges encountered in cell segmentation for each tissue type based on specific cellular features. Finally, we computed the mean value of the aforementioned 4 normalized cell morphology indicators. Based on these mean values, we stratified the image samples into low, medium, and high groups and further explored the correlation between the mean values of normalized cell morphology indicators and the F1 score. The results indicated a positive correlation between mean values and F1 scores (Fig. 4F). This finding suggests that the 4 selected indicators may, to some extent, reflect the difficulty of cell segmentation tasks.

#### Evaluation of image quality factors on model segmentation performance

Morphological metrics are used to assess the impact of biological factors on samples, and this experiment investigated the influence of image quality factors. Many deep learning models incorporate gradient information into their loss functions, such as the flow field in Cellpose1. The objective of this experiment is to investigate the impact of cell image gradients on model segmentation performance. The results demonstrate that, with regard to CellBinDB, the gradient in cell images does, in fact, exert an influence on the outcomes of the segmentation process.

**Figure 5:**
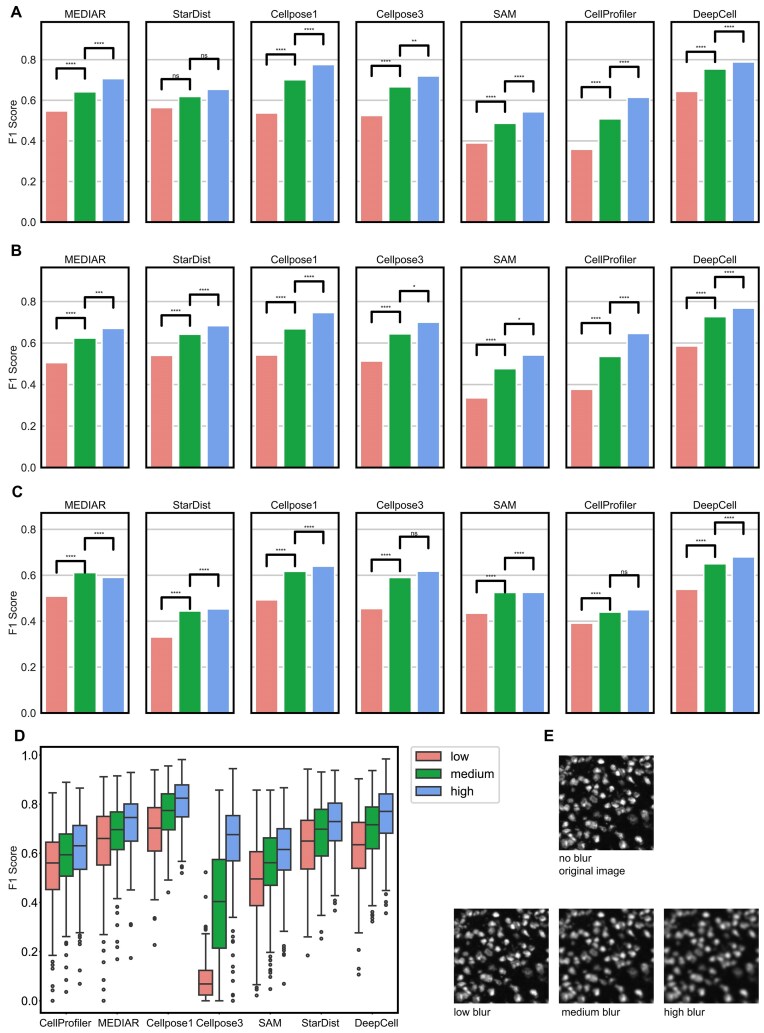
F1 score vs cell gradient groups for different algorithms. (A) DAPI-stained images, including relationship between cell gradient and F1 score. The vertical axis of the box plot is the F1 score, and the horizontal axis is the sample divided into 3 parts: low, medium, and high according to the tertile of the cell gradient. Significance mark line and *P* value are added to the box plot. The number of “*” from 1 to 4 represents a *P* value less than 0.05, 0.01, 0.001, and 0.0001. (B) Same as panel A, with results on ssDNA-stained images. (C) Same as panel A, with results on H&E-stained images. (D) Simulation experiment results. (E) examples of low, medium, and high Gaussian blurred images.

The relationship between the image gradient magnitude calculated by the Sobel operator and the F1 score shows that most algorithms perform better on cell images of a high-gradient magnitude, while their performance on low-gradient magnitude images deteriorates significantly (Fig. 5A–C). This suggests that high-gradient magnitude images often provide more edge information, facilitating cell segmentation models to perform better in accurately locating cell boundaries. Meanwhile, we also observed that the performance difference between high-gradient and medium-gradient images is relatively small. This suggests that gradient variation may not be the sole determining factor in segmentation performance, with other factors such as noise and the complexity of cell morphology potentially playing a significant role. It can therefore be concluded that a higher-gradient magnitude is beneficial to cell segmentation models, as they assist in edge detection and feature extraction.

To validate the above conclusions, we conducted simulation experiments and applied varying degrees of Gaussian blur to ssDNA images to reduce the gradient magnitude (which are detailed in the Methods section). We generated cell images with different gradient characteristics using this method (Fig. 5E) and grouped them into low, medium, and high gradient categories. Next, we applied all algorithms to segment the cells in these images and calculated the F1 scores for each algorithm across the different gradient groups. As shown in Fig. 5D, the results indicated that for all algorithms, the low, medium, and high gradient groups corresponded to low, medium, and high F1 scores, respectively. This means that high-gradient cell images, with clearer edge details, generally yielded better segmentation outcomes, while low-gradient images were more difficult to segment, leading to lower F1 scores. These experimental results further validate our previous conclusions—namely, that high-gradient cell images are easier to segment. By reducing gradients of the images, simulation of varying degrees of edge information loss observed a reduction of segmentation accuracy in low-gradient images. This demonstrates the critical role of gradients in cell segmentation.

## Discussion

In this article, we introduce CellBinDB, a comprehensive and large-scale multimodal annotated dataset designed to enrich the current cell segmentation data ecosystem and advance the development of universal cell segmentation models.

Based on CellBinDB, we evaluated the performance of cell segmentation models on a series of benchmark tasks. CellBinDB is entirely new and unseen for all the evaluated models. We first evaluated the robustness and generalization of the cell segmentation models on the entire dataset. Our results show that without retraining and fine-tuning, the non–deep learning method CellProfiler has limited generalization ability, and its performance significantly lags behind deep learning models. In stark contrast, deep learning models, especially Cellpose3, show excellent segmentation and generalization performance, highlighting their superiority in this field. Considering the significant difference in different staining types and image features learned by various models from training set, we then evaluated the cell segmentation models on image subsets of 4 staining types separately. In this evaluation, we found the challenges of cell segmentation on H&E-stained images and mIF-stained images and subsequently proposed a corresponding solution. Based on the above cell segmentation tasks, we conducted a comprehensive evaluation of the model performance and provided model selection suggestions for different cell segmentation scenarios.

Subsequently, we explore the factors that affect cell segmentation results. First, from the biological factors of the samples, differences in cell morphology, such as cell area, cell density, and roundness, directly affect the difficulty of cell segmentation. We used 4 indicators that were positively correlated with F1 score to quantify this effect. Different tissue types are directly related to cell morphology. We provide the normalized scores of cell morphological indicators for each tissue in CellBinDB to quantify the difficulty of cell segmentation in different tissues. Through this evaluation, we can estimate the performance of the model by the tissue type of the segmented image. For example, we can predict that cell segmentation in tissues, such as the small intestine, ganglion, and tongue, will be challenging. Finally, the evaluation of the impact on image quality shows that high image gradient magnitude facilities cell segmentation. The error factors such as image noise and blur will affect image gradient, which is possibly due to the limitations of the optical system itself, the sample preparation process, and the uncertainty of imaging conditions. Therefore, laboratory personnel should strictly perform experimental operations to provide higher-quality image data for downstream systems.

We fine-tuned the models participating in the benchmark on CellBinDB and subsequently assessed their performance on independent third-party datasets containing previously unseen images. The fine-tuned models show performance improvements, validating CellBinDB’s efficacy as a valuable resource for advancing cell segmentation research.

Acknowledging the substantial challenges associated with cellular image data acquisition in this domain, we have made available the 9 public datasets referenced in this study, maintaining their original formats to ensure compatibility and facilitate future research endeavors.

While this work has made a contribution, it is important to acknowledge its limitations. The dataset utilized in this study is confined to 2-dimensional (2D) images despite the variety of images. In recent years, the popularity of 3-dimensional (3D) images has increased, posing new and unique challenges for cell segmentation tasks. To address these emerging challenges, researchers have already developed algorithms capable of performing 3D cell segmentation. Acknowledging the importance of this advancement, we intend to delve into this direction and explore the potential of incorporating 3D images into CellBinDB in future research endeavors.

In conclusion, compared to any previous work, this work makes a contribution in the diversity and scale of the dataset, offering a comprehensive and extensive dataset in this work that serves as a valuable resource for advancing cell segmentation models. Nevertheless, this represents only a small step forward in the direction of universal model development. Given the complexities and nuances in the field, ongoing research and continuous expansion of datasets encompassing various staining types, tissue types, and imaging modalities are necessary. By continuing to build upon this preliminary progress, we can collectively work toward the ultimate goal of developing models that can generalize and adapt to diverse datasets, staining techniques, and tissue types in the field of cell segmentation.

## Methods

The code library of this study is implemented in Python3 [33], using numpy [34], scipy [35], pandas [36], skimage [37], opencv [38], and sklearn [29], and jupyter [39] was used to create a notebook version. The figures in this article were plotted using matplotlib [40] and seaborn [41] and formatted using Adobe Illustrator.

### Data collection and annotation

#### Image acquisition

CellBinDB includes 29 mouse tissues, 1 rat tissue, and 5 human tissues. The 5 different human tissue samples are from the open platform 10x Genomics, with download links provided in the Data Availability section. For 2 IF-stained images (human skin melanoma and human prostate cancer), only the DAPI channel was selected. The tissues used in this study were provided by the Guangzhou Institutes of Biomedicine and Health, Chinese Academy of Sciences, and the BGI laboratory. Mouse tissues were obtained from 6-week-old C57BL/6 female and male experimental mice. Rat tissues were obtained from 10-week-old male SD (Sprague-Dawley) rats. All experimental protocols for generating the dataset adhered to ethical regulations regarding animal research. Fresh-frozen samples were prepared and sectioned according to the STOmics Stereo-seq Transcriptomics Set User Manual. Tissue slices of 10 $\mu m$ were attached to the chip surface and stained after the tissue fixation. During the imaging stage, the epi-bright field (color camera) mode was selected for H&E-stained tissues while the epi-fluorescence mode was selected for fluorescent-stained tissues. After completing the photography according to the manual requirements, the imageQC module of the StereoMap software was used for image quality control.

Whole-slide images (WSIs) were generated by (i) a STOmics Microscope Go Optical equipped with Scanner Version 1.2.2, using 10×/0.75 NA and 20×/0.5 NA objective and Go Optical Scanner Ximea Mc124 for grayscale images and Go Optical Scanner Ximea Mc050 for RGB images, and (ii) a Motic PA53 FS6 microscope equipped with PA53Scanner 1.0.0.14, using 10×/0.75 NA objective and PA53 FS6 SCAN S5LITE MONO for grayscale images and PA53 FS6 SCAN S5LITE for RGB images. We obtained WSIs from the laboratory or open platforms and then cropped the size to 512 × 512 pixels or 256 × 256 pixels. A biologist selected the most representative fields of view (FOVs) for each WSI, ensuring that the selected FOV images were clear and suitable for creating ground truth.

#### Image annotation

We trained a model on CellBinDB and a team of professional annotators to semiautomatically annotate the dataset proposed in this study through 3 steps: model segmentation, manual modification or reannotation, and expert verification. The model we used for annotation was from Li et al. [42]. The model architecture was psaUnet, which contained 5 encoder blocks and 5 decoder blocks. In the preprocessing stage, median filtering was used to smooth the possible noise in the input image. In the postprocessing stage, operations such as corrosion, dilation, and watershed were performed. The preliminary results of the model segmentation were saved in Tagged Image File Format (TIFF) and then converted to JavaScript Object Notation (json) format and imported into Qupath 0.4.3 software [43]. In Qupath 0.4.3, the nuclei annotated by the model were visualized as polygons overlaid on the original nucleus images. If the model segmentation results were satisfactory, the annotation team obtained more accurate annotations by refining polygon outlines, deleting or adding polygons to modify the results of the model segmentation. Conversely, the annotation team reannotated manually. The model we trained did not support cell membrane segmentation; therefore, mIF-stained images had to be annotated manually. Meanwhile, the model annotation step was skipped, and the annotation team directly used Qupath 0.4.3 software to outline the cells with the polygon tool. These annotations were subsequently checked by experts. The incorrectly annotated images and corresponding modification suggestions were returned to the annotation team. Once the annotations were approved, the 2 types of masks (instance mask and semantic mask) were exported and converted into TIFF. Finally, the microscope images were added to the dataset along with the 2 forms of masks. Supplementary Table S2 provides detailed information, such as the file name and staining type, tissue type, size, and data source for each image.

The annotation of a cell is difficult due to issues such as being out-of-focus or nuclei exhibiting altered morphology during slide preparation. We defined the following criteria to annotate images:

- Naked eye is the highest standard, and all cells that can be identified by the naked eye are annotated ensuring no false negatives (FNs) or false positives (FPs).
- The entire cell must be completely covered without any omissions or obvious gaps.
- The annotated cell boundaries should be smooth without excessive jagged edges.
- Overlapping cells should be annotated separately.
- The segmentation should not be excessively fragmented. For elongated cells, do not split them into multiple individuals.

### Model architecture and default parameters

SAM [25] claims that it has the capability for zero-shot generalization of new image distributions and tasks. The model_type of SAM utilizes “vit_b” and applies SamAutomaticMaskGenerator to automatically generate masks without the need for external prompts.

Cellpose1 [5] can segment multiple types of cells without requiring parameter adjustments, new training data, or further model retraining. Cellpose1 uses the “cyto” model, with the channels for grayscale images set to [0,0] and [1, 3] for H&E images. The diameter is set to None.

Cellpose3 [6] introduced a novel method for biological images that achieves efficient segmentation without the requirement for clean images. Cellpose3 uses the “cyto3” model, with the channels for grayscale images set to [0,0] and [1, 3] for H&E images. The diameter is set to None, and the “denoise_cyto3” model is used for noisy images.

DeepCell [7] achieves human-level accuracy across a variety of tissues and imaging modalities while requiring no manual parameter tuning for the end user. DeepCell uses the “Mesmer” model, with image_map set to 0.5 and compartment set to “nuclear.”

MEDIAR [26] stood out in the NeurIPS 2022 cell segmentation competition [24] and achieved SOTA. MEDIAR uses the provided from_phase2.pth model, with model_args configured as follows: “classes” is set to 3, “decoder_channels” is set to [1024,512, 256,128, 64], “decoder_pab_channels” is set to 256, “encoder_name” is set to “mit_b5,” and “in_channels” is set to 3. The algo_params has “use_tta” set to True.

StarDist [9] uses star-convex polygons to represent cell shapes, allowing accurate cell localization even under challenging conditions. StarDist uses the “2D_versatile_he” model for HE images and the “2D_demo” model for non-HE images.

Cellprofiler [8] is one of the earliest high-throughput cell image analysis platforms. The Cellprofiler segmentation pipeline for non-H&E images is as follows: first, use IdentifyPrimaryObjects to identify cell objects, setting “Discard objects touching the border of the image” to No. Then, apply OverlayOutlines to outline the cells. Next, use ExpandOrShrinkObject to erode the cell boundaries, separating closely adjacent cells. MaskImage is utilized to convert the image to a mask, and the image is saved with SaveImage. For H&E images, the segmentation pipeline is similar, but it starts with an additional step: convert the image to grayscale and then invert the colors before proceeding with the same steps as for non-H&E images.

HoVerNet [30] is a novel convolutional neural network that enables simultaneous segmentation and classification of nuclei in histology images spanning multiple tissue types. We employed the default parameters provided by the official implementation, using both the tile and original modes. The settings were as follows: nr_types=0, nr_inference_workers=8, nr_post_proc_workers=16, and batch_size=32. Additionally, we utilized the CPM-17 checkpoint model weights released by the official source.

### Fine-tuning pipeline

For Cellpose and Cellpose3, we conducted fine-tuning using the cyto and cyto3 pretrained weights, respectively, in accordance with the official Cellpose 2 documentation. The fine-tuning process employed default hyperparameters and was performed on the complete set of images from the CellbinDB dataset.

For MEDIAR, we utilized the finetuning1.json configuration file provided in the official fine-tuning procedure and similarly used the entire CellbinDB dataset.

Following fine-tuning, we evaluated all 3 models on the H&E-stained dataset IEEE_TMI_2019. The results indicated that the fine-tuning process enhanced the H&E segmentation performance of all 3 models.

### Benchmark pipeline

Different from some previous work [27, 44–46], we designed a series of benchmarks to test the performance of each model without retraining. The method maximized the diversity and breadth of CellBinDB to evaluate the generalizability and robustness of each model. Unless otherwise specified, default parameters will be used. Each step of the benchmark targets a different cell segmentation scenario:

1. Model evaluation on the entire dataset. First, we test each model on the entire dataset, including all staining types and tissue types, and evaluate the overall performance results using the metrics precision, recall, F1 score, and Dice. The step aims recommend the best model for users segmenting a multimodal dataset, especially if they are unfamiliar with their data’s attributes.
2. Model evaluation on different staining types. In this step, the dataset is classified into 4 staining types, and the performance of each model is tested individually on each single staining type. This step is intended to furnish users with model recommendations for cell segmentation reliant on specific stain types, as well as to facilitate a comparison of the relative challenges imposed by different staining types.
3. Evaluating the impact of cell morphology on the performance of cell segmentation models. To quantify cell morphology, we measured a series of metrics for each cell in the dataset, which represent different cell characteristics. We then selected several metrics (cell area, average distance, cell circularity, and cell compactness) with the greatest impact on the F1 score to explore the relationship between cell morphology and model performance. Initially, the values of these 4 indicators were computed for each cell, and the mean value for each image was determined. Subsequently, the images were categorized into 3 groups—low, medium, and high—and based on tertiles to assess whether there are significant differences in the F1 scores across these groups. Given the similarity in features between DAPI- and ssDNA-stained images, these 2 types were analyzed together. In contrast, H&E-stained images were analyzed separately, and mIF-stained images were excluded from this experiment due to their unique membrane staining characteristics.
4. Exploring the effect of cellular image gradients on model segmentation performance. First, we matched individual cells with ground truth and predicted results. Then, we calculated the gradient magnitude of each cell using the Sobel operator in OpenCV, along with its corresponding F1 score. Subsequently, cells were categorized into 3 groups—low, medium, and high gradients—to assess whether there were significant differences in F1 scores across these groups.

### Metrics

#### Segmentation benchmark

To evaluate the models’ performance on the benchmark pipeline, an evaluation protocol was used, which is calculated in several steps. First, the overlap between each prediction and its closest ground-truth object is quantified using the IoU as follows:


\begin{eqnarray*}
IoU = \ \frac{{P \cap G}}{{P\cup G}}
\end{eqnarray*}

where *P* is prediction, and *G* is ground truth. If the IoU between the prediction and the closest ground-truth object is greater than 0.5, the ground-truth object is considered correctly segmented. For all ground-truth objects, the segmentation performance is then quantified using the *precision* and *recall* metrics given as follows:


\begin{eqnarray*}
\textit{precision} = \ \frac{{TP}}{{TP + FP}}
\end{eqnarray*}

\begin{eqnarray*}
\textit{recall} = \ \frac{{TP}}{{TP + FN}}
\end{eqnarray*}

where *TP* is the number of true positives, *FP* is the number of false positives, and *FN* is the number of false negatives. The F1 score is a metric used to assess the balance between precision and recall. It is the harmonic mean of precision and recall, providing a single score that encapsulate the characteristics of both parameters:


\begin{eqnarray*}
F1\ \textit{score} = 2\ \times \ \frac{{\textit{precision}\ \times \textit{recall}}}{{\textit{precision} + \textit{recall}}}
\end{eqnarray*}

In addition, a pixel-level evaluation metrics is introduced:


\begin{eqnarray*}
\textit{dice} = \ \frac{{2\ \left| {A\ \cap \ B} \right|}}{{\left| A \right| + \ \left| B \right|}} = \ \frac{{2\ \times \ TP}}{{2\ \times TP\ + \ FP\ + FN\ }}
\end{eqnarray*}

We propose to also use another metric that can be accurately quantified and interpreted to evaluate the performance of kernel instance segmentation, the panoptic quality (PQ) of kernel instance segmentation [47], defined as


\begin{eqnarray*}
PQ = \ \frac{{\left| {TP} \right|}}{{\left| {TP} \right| + \ \frac{1}{2}\left| {FP} \right| + \ \frac{1}{2}\left| {FN} \right|}}\ \times \ \frac{1}{{\left| {TP} \right|}}\mathop \sum \limits_{\left( {x,y} \right)\in TP} IoU\left( {x,y} \right)
\end{eqnarray*}

#### Cytomorphological indicators

To explore the impact of cell morphology on segmentation performance, we measured a series of morphological metrics for each cell in the dataset and selected the ones with the greatest impact, including cell area, average cell distance, cell circularity, and cell compactness.

The cell area is the number of pixels contained in each cell. The average cell distance refers to the average distance between each cell and the center point of the nearest cell. Cell circularity is used to calculate the circularity of the cell shape. The closer the value is to 1, the closer the cell shape is to a circle. We used the cv2.convexHull() function in OpenCV to get the convex hull of each cell, used the cv2.arclength function to get the convex hull area, and then calculated the circularity according to the following formula:


\begin{eqnarray*}
\textit{Circularity} = \ \frac{{4\pi \ \times \textit{Area}}}{{\textit{Convex}.{P^2}}}
\end{eqnarray*}

Compactness is the degree of compactness of the cell shape, calculated based on the cell area and cell perimeter. The closer the compactness is to 1, the more compact and circular the cell shape is; the closer the compactness is to 0, the more irregular and scattered the cell shape is:


\begin{eqnarray*}
\textit{Compactness} = \ \frac{{4\pi \ \times \textit{Area}}}{{{P^2}}}
\end{eqnarray*}

The difference between the above 2 indicators is that cell roundness uses the perimeter of the cell convex hull, while compactness uses the cell perimeter. If there are many irregular protrusions or depressions on the edge of the cell, the value of compactness will obviously deviate from 1. Circularity pays more attention to the roundness of the overall shape and is less affected by slight irregularities on the edge.

In order to more intuitively display the association between cell morphology indicators and tissue types, the above 4 morphology indicators need to be normalized before drawing the heatmap, so that indicators of different units or magnitudes can be compared and weighted. The commonly used method is linear transformation, which is to scale the data to a specific range [0, 1]. The calculation formula is as follows:


\begin{eqnarray*}
x^{\prime} = \frac{{x - {x_{min}}}}{{{x_{max}} - {x_{min}}}}
\end{eqnarray*}

where *x* is the original value. *x*_min_ and *x*_max_ are the minimum and maximum values of the metric in the entire dataset, respectively, and *x*′ is the normalized value.

#### Image gradient calculation

In image processing, gradient magnitude is an important indicator to measure the rate of change of pixel intensity in an image. It plays a key role in tasks such as edge detection. To calculate the gradient magnitude, we use the Sobel operator, which is one of the most important operators for image edge detection. The Sobel operator approximates the gradient values of the image in the horizontal (*G_x_*) and vertical (*G_y_*) directions through two 3 × 3 convolution kernels. These 2 convolution kernels perform convolution operations on the image separately to highlight the changes along their respective axes. Specifically, the Sobel operator uses the weighted difference of neighboring pixels to emphasize these changes.

We used the ground-truth mask to identify the cell regions in the cell images, then applied the Sobel operator from OpenCV (cv2) to compute the *x* and *y* gradients of the cell images, followed by calculating the gradient magnitude and average.

The formula for the gradient in the *x*-direction (*G_x_*):


\begin{eqnarray*}
{G_x} = I*\ {S_x}
\end{eqnarray*}

where *I* is the input image, and *S_x_* is the Sobel kernel matrix for the *x*-direction:


\begin{eqnarray*}
{S_x} = \ \left[ {\left. {\begin{array}{@{}*{3}{c}@{}} { - 1}&0&1\\ { - 2}&0&2\\ { - 1}&0&1 \end{array}} \right]} \right.
\end{eqnarray*}

The formula for the gradient in the *y*-direction (*G_y_*):


\begin{eqnarray*}
{G_y} = I*\ {S_y}
\end{eqnarray*}

where *S_y_* is the Sobel kernel matrix for the *y*-direction:


\begin{eqnarray*}
{S_y} = \ \left[ {\left. {\begin{array}{@{}*{3}{c}@{}} { - 1}&{ - 2}&{ - 1}\\ 0&0&0\\ 1&2&1 \end{array}} \right]} \right.
\end{eqnarray*}

The gradient magnitude (*G*) is calculated as


\begin{eqnarray*}
G = \ \sqrt {G_x^2 + \ G_y^2}
\end{eqnarray*}

It combines the gradient information in 2 directions and provides a scalar value that is proportional to the edge strength of each point in the image. In short, higher gradient magnitude values usually indicate significant edge locations in the image, which is crucial for cell segmentation tasks.

The average gradient magnitude is calculated as


\begin{eqnarray*}
\textit{Average}\ \textit{Gradient}\ \textit{Magnitude} = \ \frac{1}{N}\mathop \sum \limits_{i = 1}^N {G_i}
\end{eqnarray*}

where *N* is the number of pixels in the image, and *G_i_* is the gradient magnitude of each pixel.

### Simulation experiments

We used several different Gaussian blur parameters and adjusted the gradients of these blurred images to create 3 categories: low, medium, and high gradients. Each category of images had varying levels of blur, aimed at simulating images with different gradient magnitudes in order to assess the impact of gradient on cell segmentation performance. The blurring was implemented using OpenCV’s GaussianBlur function. The parameters for low-intensity blurring were a (5,5) kernel size with sigmaX set to 1; for medium intensity, a (9,9) kernel size with sigmaX set to 2; and for high intensity, a (13,13) kernel size with sigmaX set to 3. After applying the blur, we calculated the average gradient magnitude of the images. The calculation formula for Gaussian blurring is as follows:


\begin{eqnarray*}
\textit{Iblur}\left( {x,y} \right) = i = - k\mathop \sum \limits^k j = - k\mathop \sum \limits^k I\left( {x + i,y + j} \right) \cdot G\left( {i,j} \right)
\end{eqnarray*}

where *I*(*x* + *i, y* + *j*) is the original pixel intensity at position (*x* + *i, y* + *j*), and *G*(*i, j*) is the Gaussian kernel weight for the position (*i, j*) relative to the center.

## Availability of Source Code and Requirements

Github project homepage: https://github.com/STOmics/cs-benchmark.

Programming language: Python

Other requirements: Python 3.8 or higher

License: MIT License

RRID:SCR_026740

BiotoolsID: cellbindb

Software Heritage PID [48]: https://archive.softwareheritage.org/swh:1:snp:4248eacf2a6512e456b43bb9c9adc8f140d41051.

## Supplementary Material

giaf069_Supplemental_Files

giaf069_Authors_Response_To_Reviewer_Comments_original_submission

giaf069_GIGA-D-24-00566_original_submission

giaf069_GIGA-D-24-00566_Revision_1

giaf069_Reviewer_1_Report_Original_Submission Jeff Rhoades -- 1/22/2025

giaf069_Reviewer_1_Report_Revision_1 Jeff Rhoades -- 5/5/2025

giaf069_Reviewer_2_Report_Original_Submission Shan Raza -- 2/4/2025

giaf069_Reviewer_2_Report_Revision_1 Shan Raza -- 4/22/2025

## Data Availability

CellBinDB has been deposited into CNGB Sequence Archive (CNSA) of China National GeneBank DataBase (CNGBdb) with accession number CNP0006370: https://db.cngb.org/search/project/CNP0006370/. We have also uploaded CellBinDB and the 9 public datasets mentioned in Fig. 1E to Zenodo [49]. In addition, we uploaded 844 images of mouse and rat from CellBinDB to the BioImage Archive [50]. All additional supporting data are available in the *GigaScience* repository, GigaDB [51]. DOME-ML annotations are available in DOME registry [52]. The 9 public datasets are publicly available [53–61]. The raw data of 5 human samples from 10x Genomics are available free of charge. The download links are as follows: H&E human lung cancer: https://www.10xgenomics.com/datasets/preview-data-ffpe-human-lung-cancer-with-xenium-multimodal-cell-segmentation-1-standard. H&E human pancreas: https://www.10xgenomics.com/datasets/ffpe-human-pancreas-with-xenium-multimodal-cell-segmentation-1-standard. H&E human ovarian cancer: https://www.10xgenomics.com/datasets/ffpe-human-ovarian-cancer-data-with-human-immuno-oncology-profiling-panel-and-custom-add-on-1-standard. DAPI human skin melanoma: https://www.10xgenomics.com/datasets/human-melanoma-if-stained-ffpe-2-standard. Human DAPI human prostate cancer: https://www.10xgenomics.com/datasets/human-prostate-cancer-adjacent-normal-section-with-if-staining-ffpe-1-standard.

## Additional Files

**Supplementary Fig. S1**. Performance comparison before and after fine-tuning. (a) Comparison of F1 scores of Cellpose1, Cellpose3, and MEDIAR on the IEEE_TMI_2019 dataset before and after fine-tuning on CellBinDB. (b) Comparison of F1 scores of Cellpose1, Cellpose3, and MEDIAR on the Lizard dataset before and after fine-tuning on CellBinDB. (c) Examples of original images, ground truth, and segmentation results of Cellpose1, Cellpose3, and MEDIAR before and after fine-tuning for the IEEE_TMI_2019 and Lizard datasets.

**Supplementary Fig. S2**. Fluorescent staining and H&E staining images (except for the models shown in Fig. 4). Four indicators (cellArea, averageDistance, cellCircularity, cellCompactness) are used to evaluate the effect of cell morphology on segmentation performance. The vertical axis of the box plot is the F1 score, and the horizontal axis is the classification of samples into low, medium, and high parts according to the tertiles of each indicator. Significant marker lines and *P* values are added to the box plot. “*” from 1 to 4 indicates that the *P* value is less than 0.05, 0.01, 0.001, and 0.0001.

**Supplementary Table S1**. Summary of dataset composition in CellBinDB, including staining methods, species, data sources, tissue types, tile dimensions, and sample quantities.

**Supplementary Table S2**. Detailed metadata for each image in CellBinDB, encompassing file identifiers, annotation masks, image specifications, biological attributes, and acquisition parameters.

## Abbreviations

DAPI: 4′,6-diamidino-2-phenylindole; FOV: field of view; H&E: hematoxylin and eosin; IoU: intersection-over-union; mIF: multiplex immunofluorescence; ssDNA: single-stranded DNA; WSI: whole-slide image.
