# Supplementary material for: CellBinDB: a large-scale multimodal annotated dataset for cell segmentation with benchmarking of universal models
Source: Gigascience. 2025 Jun 24;14:giaf069. doi: 10.1093/gigascience/giaf069 (PMC12206155; doi:10.1093/gigascience/giaf069)
Supplement: giaf069_Supplemental_Files [file giaf069_supplemental_files.zip › Supplementary Figure 2.pdf]

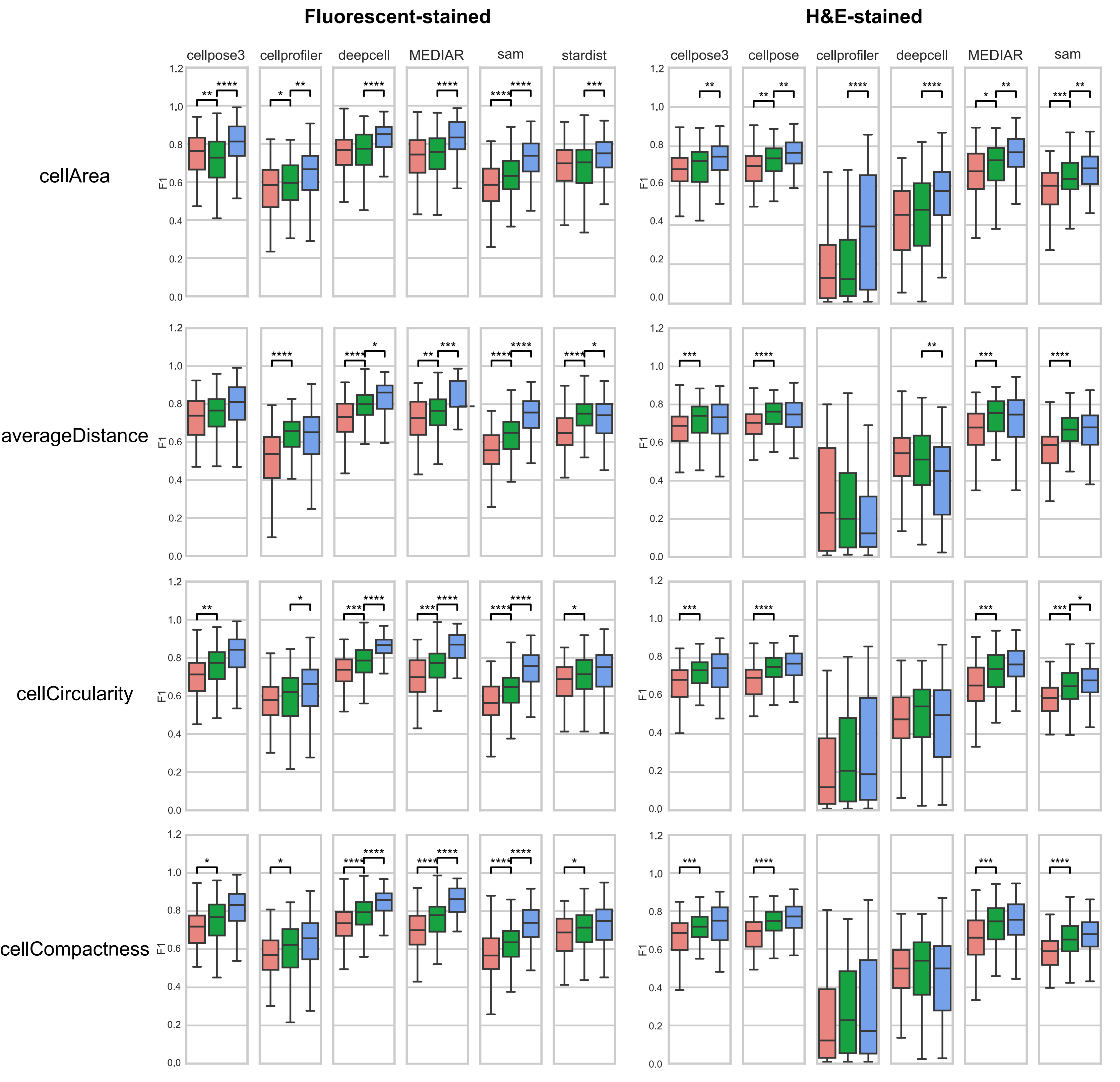

Fluorescent-stained
H&E-stained
cellpose3
cellprofiler
deepcell
MEDIAR
sam
stardist
cellpose3
cellpose
cellprofiler
deepcell
MEDIAR
sam
cellArea
averageDistance
cellCircularity
cellCompactness
F1
