# Supplementary figures and images for: CellBinDB: a large-scale multimodal annotated dataset for cell segmentation with benchmarking of universal models
Source: Gigascience. 2025 Jun 24;14:giaf069. doi: 10.1093/gigascience/giaf069 (PMC12206155; doi:10.1093/gigascience/giaf069)

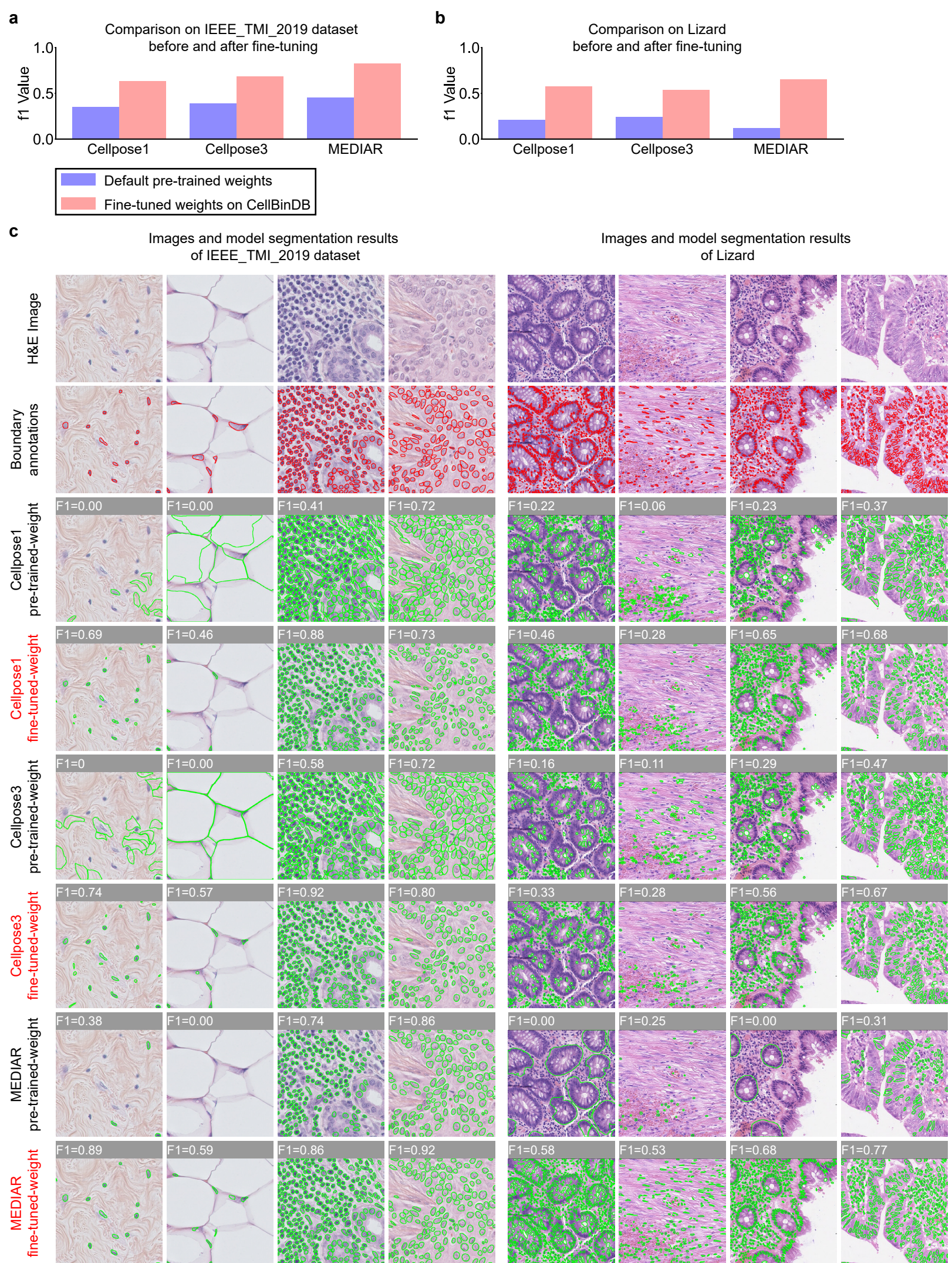

Supplement: giaf069_Supplemental_Files [file giaf069_supplemental_files.zip › Supplementary Figure 1.pdf]
