# Supplementary material for: CellBinDB: a large-scale multimodal annotated dataset for cell segmentation with benchmarking of universal models
Source: Gigascience. 2025 Jun 24;14:giaf069. doi: 10.1093/gigascience/giaf069 (PMC12206155; doi:10.1093/gigascience/giaf069)
Supplement: giaf069_GIGA-D-24-00566_Revision_1 [file giaf069_giga-d-24-00566_revision_1.pdf]

GigaScience

CellBinDB: A Large-Scale Multimodal Annotated Dataset for Cell Segmentation with Benchmarking of Universal Models

--Manuscript Draft--

**Manuscript Number:**	GIGA-D-24-00566R1
**Full Title:**	CellBinDB: A Large-Scale Multimodal Annotated Dataset for Cell Segmentation with Benchmarking of Universal Models
**Article Type:**	Research
**Funding Information:**	National Key R&D Program of China (2022YFC3400400) — Not applicable
**Abstract:**	In recent years, cell segmentation techniques have played a critical role in the analysis of biological images, especially for quantitative studies. Deep learning-based cell segmentation models have demonstrated remarkable performance in segmenting cell and nucleus boundaries, however, they are typically tailored to specific modalities or require manual tuning of hyperparameters, limiting their generalizability to unseen data. Comprehensive datasets that support both the training of universal models and the evaluation of various segmentation techniques are essential for overcoming these limitations and promoting the development of more versatile cell segmentation solutions. Here, we present CellBinDB, a large-scale multimodal annotated dataset established for these purposes. CellBinDB contains more than 1,000 annotated images, each labeled to identify the boundaries of cells or nuclei, including 4',6-Diamidino-2-Phenylindole (DAPI), Single-stranded DNA (ssDNA), Hematoxylin and Eosin (H&E), and Multiplex Immunofluorescence (mIF) staining, covering over 30 normal and diseased tissue types from human and mouse samples. Based on CellBinDB, we benchmarked eight state-of-the-art and widely used cell segmentation technologies/methods, and our further analysis reveals that complex cell shapes reduce segmentation accuracy while higher image gradients improve boundary detection, offering insights for refining segmentation strategies across diverse imaging scenarios.
**Corresponding Author:**	Mei Li, Ph.D<br>BGI-Shenzhen: BGI Group<br>SHENZHEN, GD CHINA
**Corresponding Author Secondary Information:**	
**Corresponding Author's Institution:**	BGI-Shenzhen: BGI Group
**Corresponding Author's Secondary Institution:**	
**First Author:**	Can Shi
**First Author Secondary Information:**	
**Order of Authors:**	Can Shi
	Jinghong Fan
	Zhonghan Deng
	Huanlin Liu
	Qiang Kang
	Yumei Li
	Jing Guo
	Jingwen Wang
	Jinjiang Gong
	Sha Liao

Powered by Editorial Manager® and ProduXion Manager® from Aries Systems Corporation

	Ao Chen
	Ying Zhang
	Mei Li
**Order of Authors Secondary Information:**	

Response to Reviewers:

Response to Reviewers

Dear editor and reviewers,

Thank you very much for your careful evaluation and valuable feedback on our manuscript, “CellBinDB: A Large-Scale Multimodal Annotated Dataset for Cell Segmentation with Benchmarking of Universal Models” (GIGA-D-24-00566). We sincerely appreciate the constructive comments, which have helped us improve the quality and clarity of the manuscript. We have revised the manuscript accordingly, and the main changes are highlighted in red. Below, we provide a detailed, point-by-point response to each of the reviewers’ comments.

Authors Response to Comments of Editor

Comment: In addition, please register any new software application in the bio.tools and SciCrunch.org databases to receive RRID (Research Resource Identification Initiative ID) and biotoolsID identifiers, and include these in your manuscript. Computational workflows should be registered in workflowhub.eu and the DOIs cited in the relevant places in the manuscript.
Response: Thanks for your comments.
We have registered our software application on both bio.tools and SciCrunch.org, and the corresponding biotoolsID and RRID have been added to the Code availability section of the revised manuscript.
Regarding the suggestion to register our computational workflow on workflowhub.eu, we would like to clarify that our computational pipeline was developed entirely using custom Python scripts, and it does not follow a standardized workflow format (e.g., Galaxy, Nextflow, CWL) that is compatible with workflowhub.eu. Therefore, it may not be appropriate to register our code as a workflow in workflowhub.eu. However, to ensure reproducibility and facilitate sharing, we have made all source code publicly available on GitHub. This link is also available in the Code availability section of the manuscript.

Authors Response to Comments of Reviewer 1

Major Areas for Improvement:
Comment 1: Preprocessing Uniformity: Apply preprocessing steps uniformly across all segmentation approaches to ensure fair comparisons and avoid bias.
Response 1: Thanks for your suggestion.
For the mIF-stained images, we applied the preprocessing steps (color inversion) to all methods included in the benchmark. For the H&E-stained images, we applied the preprocessing steps (grayscale conversion and color inversion) to all models except Stardist and HoverNet to align their inputs with the expected data distribution. However, for Stardist and HoverNet, we intentionally avoided these preprocessing steps, as both models were specifically optimized for H&E images, and additional preprocessing could potentially shift the data away from their training distribution. For detailed modifications, see the third paragraph of the Evaluation on four staining types respectively section, and Figure 3b-e.

Comment 2: Inclusion of Cellpose3 Training Dataset: The manuscript should include the dataset used for training Cellpose3 in its comparisons. Cellpose3's superior generalist model performance is emphasized, yet the absence of its training dataset in the comparisons raises questions about robustness of the benchmarking.
Response 2: Thanks for your suggestion, we have added the training set of Cellpose3. Cellpose3 used nine publicly available datasets to train the “cyto3” model. These include Cellpose's own dataset and 8 other public datasets. Considering that the scope of this study is the microscope images of stained cells, not all the datasets used for Cellpose3 training can be added to this study. The details are as follows:
- Cellpose: Cellpose includes a wide range of images, such as fruits, vegetables,

artificial materials, fish scales, reptile scales, starfish, jellyfish, sea urchins, rocks, shells, and some bacterial microscopy images. We partially included this dataset by selecting only the microscopy images related to stained cells. Non-microscopy images and bacterial samples were excluded to maintain focus.
•Omnipose, YeaZ, DeepBacs, LiveCell: were not included in the study. These datasets primarily feature images of bacteria, yeast, nematodes, plants, or were captured using phase contrast microscopy, which are not aligned with our focus on stained cell microscopy images.
•TissueNet, MoNuSeg, BBBC038, BBBC039: These datasets were previously included in the study because they contained microscopy images of stained cells that were highly consistent with our research goals.
Therefore, we only newly added the Cellpose dataset, which is described in the second paragraph of Introduction section, Table 1, and Figure 1e.

Comment 3: Evidence of Dataset Utility: While the dataset's benchmarking is well-done, the manuscript does not provide evidence that models trained on CellBinDB outperform those trained on other datasets. Addressing this, though potentially out of scope, would strengthen the manuscript's impact.
Response 3: We sincerely appreciate the reviewer's valuable suggestion regarding the comparative analysis of models trained on CellBinDB versus other datasets. While a direct comparison with models trained on alternative datasets would indeed be insightful, we believe that our current experiments already provide evidence of CellBinDB's utility in a different but equally meaningful way.
Specifically, we conducted fine-tuning experiments to evaluate whether models pre-trained on CellBinDB can improve performance on unseen datasets (details are provided in the last paragraph of the Introduction, the Fine-tuning pipeline section in Methods, the fourth paragraph of the Discussion, and Supplementary Figure 1). The results demonstrate that models initialized with CellBinDB exhibit enhanced generalization ability, achieving superior performance compared to baseline models trained from scratch. This indicates that CellBinDB serves as a high-quality pre-training resource, facilitating better adaptation to new data distributions—a key aspect of dataset utility in real-world applications.
While we acknowledge that a direct head-to-head comparison with other datasets could further strengthen our claims, such an analysis would require extensive benchmarking across multiple existing datasets, which is beyond the scope of this work focused on establishing and validating CellBinDB as a standardized benchmark. Nevertheless, we fully agree that future studies (including our own) could explore this direction, and we hope the current fine-tuning results—along with the dataset's diversity, scale, and annotation quality—sufficiently support its value to the community. Thank you for your constructive feedback, which has helped us better highlight CellBinDB's practical relevance.

Comment 4: Figure Panels:
* Labeling in figure panels should be clearer to enhance interpretability. For instance, indicate whether the instance or semantic masks are being shown and consider making instance segmentation masks colorful to highlight unique IDs.
* Semantic masks could be omitted if space is constrained, as they are largely redundant with instance masks.
* Ensure figures are spaced more evenly throughout the text, ideally located near their first references, to improve readability.
Response 4: Thank you for pointing out the problem with the figure panel in our article. We reviewed all figure panels and revised unclear labels. In particular, we removed the semantic masks in Figure 1c because they are redundant with instance masks, and added color coding to instance masks to highlight unique IDs. In addition, we split the original Figure 2 into Figure 2 and Figure 3 to ensure that the figures are more evenly spaced throughout the text.

Comment 5: Abstract Clarity: The abstract should better reflect the intellectual contributions of the analysis of segmentation performance factors (i.e. cell morphology and image gradients).
Response 5: Thank you for your suggestion on the Abstract section. We added relevant descriptions of cell morphology and image gradients in the abstract to make the analysis of factors affecting segmentation results more prominent. The details are at the end of the Abstract.

Powered by Editorial Manager® and ProduXion Manager® from Aries Systems Corporation

Comment 6: Normalization Methods: Provide details on how cell morphology indicators are normalized in the methods section to ensure reproducibility and clarity.
Response 6: We have provided detailed information and calculation formulas on how to normalize cell morphology metrics in the Metrics of Methods section.

Comment 7: Explanation of Image Gradient: The discussion of gradient magnitude and its calculation using the Sobel operator requires more accessible language. Not all readers will be familiar with this concept, so additional context is essential.
Response 7: We have added a more detailed introduction to the Sobel operator, combined with the specific calculation formula, hoping to help readers without relevant background to understand. The added content is included in the Cytomorphological Indicators section under Metrics of the Methods.

Comment 8: Tissue Classification: Group related tissues, such as "brain", "half brain" and "cerebellum", under a common "neural tissue" category for easier interpretation and analysis.
Response 8: Thank you for your suggestion. For ease of interpretation and analysis, some tissues can be grouped into one general category. After checking the images in CellBinDB, we believe that the image features of “brain”, “half brain” and “cerebellum” are similar, so we group them into the general category of “brain”. Other tissues, such as “large intestine” and “small intestine”, were not combined because their image features were significantly different. For related content, please see Supplementary Table 1, Supplementary Table 2, Figure 1b and Figure 4e.

Additional Suggestions:
Comment 9:
* Address grammatical errors and improve clarity in some sections, such as the benchmarking pipeline description.
* Replace vague terms like “ML-based” when referring to CellProfiler with specific algorithmic descriptions.
* Including public datasets, such as Cellpose, to create a unified, all-inclusive CellBinDB dataset might significantly enhance the resource's utility for machine learning practitioners.

Response 9: Thank you for your suggestions, which are very meaningful for improving the readability of our article. We have checked and revised the possible grammatical errors and unclear references in the manuscript, and uploaded the 9 public datasets involved in this study to our Zenodo project (https://zenodo.org/records/14312044) for the convenience of readers.

Authors Response to Comments of Reviewer 2

Comment 1: The authors missed one of the biggest data sets on the cell segmentation and classification which includes more than 500,000 annotated nuclei in H&E. The CoNIC challenge paper also analysis state-of-the-art nuclei segmentation and classification methods. The authors should add one of the best performing models in their analysis. I would also suggest the authors to include PQ and froc in the metrics to analyse the results as this is commonly used in this domain for comparison. I would also suggest to compare the results with HoVerNet or HoVerNext which are state-of-the-art algorithms for nuclei instance segmentation. The code for these algorithms is publicly available.
Response 1: Thanks for your valuable suggestions.
Regarding the dataset and the best performing model of the CoNIC challenge you mentioned, we carefully reviewed the relevant literature. As described in the CoNIC challenge publication(Graham et al., 2024), the best performing model in the CoNIC challenge is Stardist, which has been included in our analysis. The patches in CoNIC challenge dataset are extracted from the original Lizard dataset, which has already been included in our research and therefore was not added redundantly.
Regarding the addition of PQ and froc indicators you mentioned, we have carefully considered it. Some models may perform well at the pixel level (hence the high F1 score), but the segmentation of instance boundaries is not accurate enough. The PQ indicator can show this, so we added the PQ indicator, which is specifically shown in Figure 2a-e, and added a detailed description and calculation formula in the Metrics section of Methods.

	But for the froc metric, we fully understand that the froc curve is widely used in certain detection-based medical image analysis tasks, especially when evaluating the sensitivity and false positive rate of models at different thresholds. However, in our work, the main focus is on cell segmentation, not object detection or localization. Compared with detection tasks, segmentation models are designed to accurately depict individual instances at the pixel level, which makes the application of the froc curve less intuitive and less informative for such task scenarios. In order to more comprehensively evaluate the performance of the model, we supplemented the introduction of the AP-IoU curve, which can reflect the performance of the model under different degrees of strictness of matching conditions (different IoU thresholds), and can more carefully demonstrate the robustness and segmentation quality of the model. The relevant content is in the second paragraph of Results, Evaluation on four staining types respectively, Figure 2f-i. We believe that this method can more accurately reflect the actual performance of the model in the segmentation task.<br>Finally, we added the HoverNet model pre-trained on the Kumar dataset (the Kumar dataset contains various tissue type which is similar to CellBinDB). In the benchmark, we only added HoverNet to the comparison on the H&E subset and HoverNet performed well. We did not test HoverNet on other staining types of datasets because: 1. HoveNet is specifically designed for pathology images and has not been trained on fluorescent staining datasets; 2. HoveNet is not a multimodal model and is not scalable. The relevant content is in the first paragraph of Results, Evaluation on four staining types respectively, Figure 2d.<br>We hope this clarification addresses your concerns and improves the overall rigor of our evaluation.<br><br>reference<br>Graham, S. et al. (2024) ‘CoNIC Challenge: Pushing the frontiers of nuclear detection, segmentation, classification and counting’, Medical Image Analysis, 92, p. 103047. Available at: https://doi.org/10.1016/j.media.2023.103047.<br><br>Best wishes ,<br>Mei
**Additional Information:**	
**Question**	**Response**
Are you submitting this manuscript to a special series or article collection?	No
**Experimental design and statistics**<br><br>Full details of the experimental design and statistical methods used should be given in the Methods section, as detailed in our Minimum Standards Reporting Checklist. Information essential to interpreting the data presented should be made available in the figure legends.<br><br>Have you included all the information requested in your manuscript?	Yes
**Resources**<br><br>A description of all resources used, including antibodies, cell lines, animals	Yes

and software tools, with enough information to allow them to be uniquely identified, should be included in the Methods section. Authors are strongly encouraged to cite Research Resource Identifiers (RRIDs) for antibodies, model organisms and tools, where possible.<br><br>Have you included the information requested as detailed in our Minimum Standards Reporting Checklist?	
**Availability of data and materials**<br><br>All datasets and code on which the conclusions of the paper rely must be either included in your submission or deposited in publicly available repositories (where available and ethically appropriate), referencing such data using a unique identifier in the references and in the "Availability of Data and Materials" section of your manuscript.<br><br>Have you have met the above requirement as detailed in our Minimum Standards Reporting Checklist?	Yes
GigaScience has policies and guidelines in place for the use of generative AI-writing tools such as ChatGPT. If you have used such writing tools to assist with writing the manuscript this must be declared and cited in the text. Authors should not list AI-writing tools and other AI-assisted technologies as an author or co-author and should acknowledge that they are fully responsible for text generated or refined by AI-writing tools.<p><br><br>A summary of use (particularly in the introduction or among methods) needs to be included at the end of the paper, and the outputs should also be included as a supplementary file hosted in GigaDB or other open repositories. Please read our guidelines for more information. <p><br><br>By submitting to GigaScience, you are	No

aware of the journal's AI-writing tools policy, and if you have declared use of such tools below, you have acknowledged this where appropriate in your manuscript and have made a summary of use and outputs available. <p><br><b>AI-assisted writing tools have been used in the preparation of this manuscript?	

Manuscript

Click here to access/download;Manuscript;CellBinDB Revised Manuscript-20250521_clean.docx

CellBinDB: A Large-Scale Multimodal Annotated Dataset for Cell Segmentation with Benchmarking of Universal Models

Can Shi[1,2,†], Jinghong Fan[1,2,†], Zhonghan Deng[1], Huanlin Liu[1], Qiang Kang[1], Yumei Li[1], Jing Guo[1], Jingwen Wang[1], Jinjiang Gong[1], Sha Liao[1], Ao Chen[1], Ying Zhang[1,*], Mei Li[1,*]

[1] BGI Research, Shenzhen 518083, China

[2] School of Artificial Intelligence, University of China Academy of Science, Beijing 100049, China

[*]Corresponding: zhangying7@genomics.cn, limei1@genomics.cn

[†]These authors contributed equally as the first authors.

ORCID iDs:

Can Shi [0009-0007-0003-2378]; Jinghong Fan [0009-0001-9556-4831]; Zhonghan Deng [0009-0006-8474-3056]; Huanlin Liu [0009-0004-4538-5992]; Qiang Kang [0000-0001-6579-7944]; Yumei Li [0009-0007-8596-101X]; Jing Guo [0009-0004-8658-2271]; Jingwen Wang [0009-0005-9156-5350]; Jinjiang Gong [0009-0009-6529-9422]; Sha Liao [0009-0002-9358-5169]; Ao Chen [0000-0002-9699-8340]; Ying Zhang [0000-0003-3830-1338]; Mei Li [0000-0003-3310-2911]

Abstract

In recent years, cell segmentation techniques have played a critical role in the analysis of biological images, especially for quantitative studies. Deep learning-based cell segmentation models have demonstrated remarkable performance in segmenting cell and nucleus boundaries, however, they are typically tailored to specific modalities or require manual tuning of hyperparameters, limiting their generalizability to unseen data. Comprehensive datasets that support both the training of universal models and the evaluation of various segmentation techniques are essential for overcoming these limitations and promoting the development of more versatile cell segmentation solutions. Here, we present CellBinDB, a large-scale multimodal annotated dataset established for these purposes. CellBinDB contains more than 1,000 annotated images, each labeled to identify the boundaries of cells or nuclei, including 4',6-Diamidino-2-Phenylindole (DAPI), Single-stranded DNA (ssDNA), Hematoxylin and Eosin (H&E), and Multiplex Immunofluorescence (mIF) staining, covering over 30 normal and diseased tissue types from human and mouse samples. Based on CellBinDB, we benchmarked eight state-of-the-art and widely used cell segmentation technologies/methods, and our further analysis reveals that complex cell shapes reduce segmentation accuracy while higher image gradients improve boundary detection, offering insights for refining segmentation strategies across diverse imaging scenarios.

Keywords: Dataset; Cell Segmentation; Benchmark; Universal Models

Introduction

Cell/nuclear staining technology enhances the visualization of cell or subcellular resolution level in microscope images, thereby contributing to quantitative analysis in biomedical research[1]. As microscopy advances to study complex biological structures in great detail and produce rich, dense images[2], there is an urgent need for automated methods to extract cellular information by accurately segmenting cells and nuclei[1,3], especially important in spatial transcriptomics. Despite the significant progress made by deep learning methods in segmenting cell and nuclear boundaries in limited latent features of some specific images[4–14], challenges remain in developing universally applicable models. One of the key obstacles is the lack of large and diverse annotated image datasets that can support the training and evaluation of robust universal models[15–18].

Previous works have made significant contributions by releasing several public datasets for training and evaluating deep learning models, although all important advances, they are limited in their ability to simultaneously meet the requirements for large-scale, multiple staining techniques, and diverse tissue types, thus unable to fulfill the needs for universal models. Table 1 shows the annotated stained datasets that have been actively used by the research community in recent years. Some datasets are limited in scale or tissue type richness, such as MoNuSeg[15], IEEE_TMI_2019[19] and the fluorescence image dataset published by Kromp et al. in 2020[20], all of which contain fewer than 100 images. A slightly larger dataset Lizard[21] only consists of 1 tissue type. Table 1 also shows that most of the mentioned datasets are based on H&E staining, since Hematoxylin and Eosin (H&E) staining is the most common type of staining in routine pathology[22]. Followed by Immunofluorescence (IF) staining, which is more frequently employed in research settings. The Cellpose dataset contains a large number of non-microscope images of repeated objects, such as fruits, vegetables, rocks, etc., and microscope images of bacteria. When considering mammalian stained cell images, a total of 613 images were included in the comparative analysis. NuInsSeg[22] is limited to a single staining type of H&E. Similarly, BBBC039[23] is limited to Hoechst, a membrane-permeable fluorescent dye. Additionally, there are also three large-scale and more diverse datasets. The Data Science Bowl 2018 featured a dataset (BBBC038v1)[18] with 37,333 manually annotated cell nuclei, however, datasets commonly utilized for training and benchmarking segmentation models in stained cell images are still significantly larger by comparison. TissueNet[7] emphasizes immunostaining while lacking histological staining and nucleic acid staining. NeurIPS 2022 dataset[24] aims to achieve richness in image modalities, with constrained quantity in each image type, especially in the case of stained images.

Recently, many efforts have focused on developing universal methods and demonstrating robust performance on unseen datasets[5–7,9,25,26]. SAM(RRID:SCR_023680)[25], a method based on the self-attention mechanism, has the capability of Zero-shot generalization of new image distributions and tasks. Cellpose1 (RRID:SCR_021716)[5] is based on the U-Net architecture, which can precisely segment cells from diverse image types without model retraining or parameter adjustments. Cellpose3(RRID:SCR_021716)[6], an improved version, specializes in out-of-box segmentation of noisy, blurred or under-sampled images. DeepCell(RRID:SCR_022197)[7], a deep learning algorithm

for accurate whole-cell segmentation that achieves human-level segmentation performance by combining a ResNet50 backbone network and a feature pyramid network. MEDIAR[26] emerged as the state of the art (SOTA) in the NeurIPS 2022 multimodal cell segmentation competition. StarDist[9] utilizes star-convex polygons to represent cell shapes, enabling accurate cell localization even under challenging conditions, especially when dealing with overlapping cells. However, the diversity of currently available models and the inconsistency of segmentation quality metrics make it difficult to evaluate their relative performance based on literature descriptions[27]. Therefore, it is necessary to evaluate these universal algorithms on a new unseen annotated dataset.

In this study, we present a new large-scale, multimodal annotated dataset, CellBinDB, containing images of four staining types (DAPI, H&E, ssDNA, and mIF) derived from over 30 human and mouse tissues. The primary statistic of CellBinDB is presented in the last row of Table 1. Unlike previous efforts that may be limited in tissue type coverage[23,24], CellBinDB includes a wide range of both normal and diseased tissues, making it one of the most comprehensive tissue-type datasets available. The images included in the dataset were obtained from the 10x Genomics platform, as well as from new experiments based on Stereo-seq technology. Given that manual annotation is a time-intensive task, it is significantly limiting the scale of datasets. Besides, model annotation methods are affected by model style and cannot guarantee accuracy. To balance quality and efficiency in annotating this multimodal dataset, we used a combination of manual and semi-automatic annotation strategies. To eliminate potential biases in reference model[25], the model segmentation results were manually revised by a trained team of professional annotators and then checked by experts, ensuring that all annotations passed through two rounds of review. We make CellBinDB available to the research community and evaluate the performance of some general models on the dataset, providing recommendations for the selection of cell segmentation models in different scenarios. Furthermore, several cell segmentation models were fine-tuned using CellBinDB and subsequently evaluated on the independent IEEE_TMI_2019 and Lizard dataset. The fine-tuned models showed significant performance improvements (Supplementary Figure 1), and these results substantiate the utility of CellBinDB as a valuable resource for advancing model development and performance optimization in cell segmentation research.

Table.1 Publicly available annotated cell segmentation datasets.

Dataset	Staining type	Image tiles	Cell number	Organs	Tile size (pixels)
MoNuSeg[15]	H&E	44	28,846	9	1000 × 1000
IEEE_TMI_2019[19]	H&E	80	25,645	8	512 × 512 and 1000 × 1000
Kromp *et al.*[20]	IF\DAPI	79	7,813	5	550 × 430 to 1360 × 1024
Lizard[21]	H&E	238	495,179	1	512 × 512
Cellpose[5]	IF	613	about 70,000	unknown	147 × 164 to 2560 × 2160
NuInsSeg[22]	H&E	665	30,698	31	512 × 512

BBBC038v1[18]	DAPI\Hoechst \H&E	841	37,333	unknown	256 × 256
BBBC039[23]	Hoechst	200	23,165	unknown	520 × 696
TissueNet[7]	IF	3,200	1.3 million	9	512 × 512
NeurIPS 2022 cell segmentation competition dataset[24]	Jenner – Giemsa\IF	713	134,710	unknown	512 × 512 to 4096 × 4096
**CellBinDB**	**DAPI\ssDNA\H&E\mIF**	**1044**	**109,083**	**35**	**256 × 256 and 512 × 512**

Results

Dataset

In this study, we propose CellBinDB, a dataset comprising 1,044 annotated microscopy images and 109,083 cell annotations. This dataset contains four staining types: DAPI, ssDNA, H&E, and mIF (Figure 1a). CellBinDB encompasses samples derived from human and mouse species, covering over 30 histologically diverse tissue types, including disease-relevant tissues (Figure 1b). The images in CellBinDB come from two sources: 844 mouse images were from in-house experiments based on Stereo-seq technology, and 200 human images obtained from the open-access platform 10x Genomics. We annotate all images in CellBinDB and offer two types of image annotations: semantic and instance masks (Figure 1c). Annotation is a combination of manual and semi-automatic methods, with 60% manual annotation and the rest from semi-automatic annotation (Figure 1f). The annotation workflow is shown in Figure 1g, and all annotations double-checked by experts to ensure quality. Additional details are in the Methods section. CellBinDB has diverse features that make it suitable for training generalized segmentation models. To visualize the image features of CellBinDB, we applied t-distributed stochastic neighbor embedding (t-SNE[28,29]) to neural network-learned image features, revealing clusters that generally align by staining type (Figure 1d). The ssDNA and DAPI staining types show similar features, while there are significant differences between images of the same staining type from the two sources. CellBinDB demonstrates a broader feature distribution compared to existing datasets with overlapping staining types, encompassing characteristics from most major public benchmarks (Figure 1e). However, certain staining modalities (Hoechst stain in BBBC038/039) remain outside its current scope. Supplementary Table 1 provides further details about CellBinDB.

Benchmark performance

We evaluated several widely recognized segmentation models on CellBinDB, including specialized cell segmentation models: Cellpose1[5], Cellpose3[6], StarDist[9], DeepCell[7], MEDIAR[24,26], a model for general segmentation: SAM[25], and a software widely used in biomedicine and life sciences: CellProfiler(RRID:SCR_007358SAM)[8]. Among them, Cellpose1, Cellpose3, StarDist and DeepCell are models based on U-Net, MEDIAR and SAM are models based on Transformer, and

CellProfiler is a model based on machine learning. Since the models were trained on different training sets, a fair evaluation was conducted using CellBinDB, a dataset that none of the models had been trained on. The evaluation on CellBinDB was first performed on the entire dataset, followed by separate evaluations on each staining type. In H&E subset evalution, we also included HoverNet[30], a specialized model specifically designed for pathological H&E image analysis, in our comparative assessment.

Evaluation results on the entire dataset

The initial objective was to assess the capacity of each model to perform segmentation on multimodal cell images (Figure 2a). The experimental results demonstrate that most of them exhibited excellent performance except for CellProfiler and DeepCell. Of the models evaluated, Cellpose3 demonstrated the most optimal performance and was the most highly recommended (precision: 0.82, recall: 0.61, F1 score: 0.70, dice: 0.72). In contrast, the performance of Cellpose1, with a similar architectural design, was less impressive in terms of a lower F1 score than Cellpose3. It is hypothesized that this inferior performance is attributable to the smaller and less diverse training dataset employed for Cellpose1 in comparison to Cellpose3. Similarly, DeepCell demonstrated suboptimal overall performance due to the limited diversity and lack of variability in its training dataset, which consisted exclusively of fluorescent staining images. Furthermore, the machine learning-based CellProfiler model demonstrated the poorest performance among the evaluated models. In addition, compared with the F1 score, all models obtained lower PQ indexes, indicating that the models did not capture complex or fuzzy boundaries well and were not able to correctly separate closely adjacent instances, resulting in overlapping or sticky boundaries. Future model development could focus on improving the boundary accuracy of instance segmentation, enhancing the detection recall rate, and refining classification accuracy to further optimize model performance.

Evaluation on four staining types respectively

We next evaluated the segmentation capabilities of each model on four staining types respectively. Figure 1 illustrates the diverse characteristics of images in CellBinDB. The evaluation of the models on the four staining types highlights the varying levels of difficulty across the stains and the strengths of each model. In detail, most models scored higher on DAPI-stained images (Figure 2b,f,j) and ssDNA-stained images (Figure 2c,g,k). Notably, Cellpose1, DeepCell, and MEDIAR performed exceptionally well on these two stain types and are highly recommended. In contrast, significant variability was observed in model performance on H&E-stained images (Figure 2d,h,l) and mIF-stained images (Figure 2e,i,m). On H&E-stained images, StarDist, SAM, HoverNet and Cellpose3 performed well, whereas other models showed poor results or even failed. We attributed StarDist's superior performance to its specialized weights trained for H&E-stained images, the effective segmentation of HoverNet to its design specifically for H&E-stained pathology images, while SAM and Cellpose3 benefited from their large training datasets, enabling them to effectively segment H&E images. The poor performance of CellProfiler and DeepCell likely stemmed from their incorrect interpretation of the foreground and background (Figure 2l). mIF is a membrane stain that

has the opposite color characteristics compared to DAPI and ssDNA, showing that the inside of the cell is dark. On mIF-stained images, Cellpose1, Cellpose3, and MEDIAR are the most recommended. While SAM was able to process mIF images, its performance was relatively poor. The remaining models failed to effectively perform cell segmentation on mIF images because they mistakenly segmented the bright cell membranes as cells. Overall, H&E-stained and mIF-stained images pose greater segmentation challenges than other stain types.

On CellBinDB, we also matched the model's predictions to the ground-truth masks at different matching accuracy thresholds based on the standard intersection-over-union metric (IoU). We evaluateed performance using the average precision metric (AP). The above-mentioned top-performing models maintained good performance both at the commonly used IoU threshold of 0.5 and higher IoUs, which benchmark the ability to accurately segment cell boundaries.

Considering the specificity of H&E-stained image, some models perform additional preprocessing of the image, such as converting RGB to grayscale, or processing color deconvolution[31], to make the image features more consistent with the fluorescent stained image. With the exception of StarDist and HoVerNet—both specifically optimized for H&E image analysis—all six comparative models underwent standardized preprocessing involving grayscale conversion and color inversion (Figure 3a). Quantitative evaluation revealed that preprocessing substantially enhanced segmentation accuracy for the initially underperforming models, while models with baseline high performance exhibited either marginal improvements or maintained their original accuracy levels (Figure 3b, d). Among them, DeepCell exhibited the most significant performance improvement; without preprocessing, it could hardly segment H&E images. After adding preprocessing, CellProfiler and DeepCell were able to correctly interpret the foreground and background, and the performance of MEDIAR and Cellpose1 also improved to varying degrees. These results indicate that models originally designed for fluorescent images could be more effectively adapted to RGB image segmentation through preprocessing. After completing the above preprocessing, we recommended using Cellpose1, Cellpose3, or MEDIAR for cell segmentation of H&E-stained images. Similarly, this approach can be extended to mIF staining, with several previously underperforming models benefiting, particularly StarDist (Figure 3c,e), but still did not outperform Cellpose1, Cellpose3, and MEDIAR. The preprocessing process is shown in Figure 3a. Moreover, SAM demonstrates the capacity to process a range of stain types with consistent efficacy, although its performance is not as robust as that of models tailored for cell segmentation. This is believed to be due to the training data used for SAM, which is likely to contain a significant number of images that are not cells. In the absence of predefined settings or preprocessing, it is recommended to utilise SAM as a model capable of multimodal cell image segmentation.

Factors affecting cell segmentation performance

Next, we analyzed the factors that affect the model's ability to segment cells from both biological and non-biological perspectives.

Evaluation of impact of cell morphology

We evaluated a series of cell morphology metrics, including the cell morphology evaluation metrics in BIDCell[32] as well as an original metric, cell average distance, for evaluating cell density. The four metrics that exhibited the strongest correlation with the F1 score in the case of CellBinDB were found to be cell area, average distance, cell circularity and cell compactness. The results of the experiment indicate that an increase in the metrics is associated with an improvement in the performance of the segmentation. To illustrate, Cellpose1 demonstrated the most favourable performance in fluorescence staining (Figure 4a,c), whereas StarDist exhibited the most optimal results in H&E-stained images (Figure 4b,d). The full set of conclusions, which includes all models, can be found in the Supplementary Figure 2. The fluorescence staining images do not exhibit a notable distinction between the low and medium categories with respect to the indicator cellArea. This observation may be attributed to the intrinsic characteristics of the data, the cell areas of majority of images displaying cell areas concentrated within the region with lower values.

Cell morphology is directly related to tissue type. We classify the images in the dataset according to tissue type, calculate the above four indicators, and then normalize them. These results are presented in Figure 4e, which provides an overview of the difficulty level of cell segmentation for different tissue types and indicates the potential challenges encountered in cell segmentation for each tissue type based on specific cellular features. Finally, we computed the mean value of the aforementioned four normalized cell morphology indicators. Based on these mean values, we stratified the image samples into low, medium, and high groups and further explored the correlation between the mean values of normalized cell morphology indicators and the F1 score. The results indicated a positive correlation between mean values and F1 scores (Figure 4f). This finding suggests that the four selected indicators may, to some extent, reflect the difficulty of cell segmentation tasks.

Evaluation of Image Quality Factors on Model Segmentation Performance

Morphological metrics are used to assess the impact of biological factors on samples, this experiment investigated the influence of image quality factors. Many deep learning models incorporate gradient information into their loss functions, such as the flow field in Cellpose1. The objective of this experiment is to investigate the impact of cell image gradients on model segmentation performance. The results demonstrate that, with regard to CellBinDB, the gradient in cell images does, in fact, exert an influence on the outcomes of the segmentation process.

The relationship between the image gradient magnitude calculated by the Sobel operator and the F1 score shows that most algorithms perform better on cell images of high-gradient magnitude, while their performance on low-gradient magnitude images deteriorates significantly (Figure 5a,b,c). This suggests that high-gradient magnitude images often provide more edge information, facilitating cell segmentation models to perform better in accurately locating cell boundaries. Meanwhile, we also observed that the performance difference between high-gradient and medium-gradient images is

relatively small. This suggests that gradient variation may not be the sole determining factor in segmentation performance, with other factors such as noise and the complexity of cell morphology potentially playing a significant role. It can therefore be concluded that higher gradient magnitude is beneficial to cell segmentation models, as they assist in edge detection and feature extraction.

To validate the above conclusions, we conducted simulation experiments and applied varying degrees of Gaussian blur to ssDNA images to reduce the gradient magnitude (which are detailed in the Methods section). We generated cell images with different gradient characteristics using this method (Figure 5e), and grouped them into low, medium, and high gradient categories. Next, we applied all algorithms to segment the cells in these images and calculated the F1 scores for each algorithm across the different gradient groups. As shown in Figure 5d, the results indicated that for all algorithms, the low, medium, and high gradient groups corresponded to low, medium, and high F1 scores, respectively. This means that high-gradient cell images, with clearer edge details, generally yielded better segmentation outcomes, while low-gradient images were more difficult to segment, leading to lower F1 scores. These experimental results further validate our previous conclusions, namely that high-gradient cell images are easier to segment. By reducing gradients of the images, simulation of varying degrees of edge information loss observed a reduction of segmentation accuracy in low-gradient images. This demonstrates the critical role of gradients in cell segmentation.

Discussion

In this paper, we introduce CellBinDB, a comprehensive and large-scale multimodal annotated dataset designed to enrich the current cell segmentation data ecosystem and advance the development of universal cell segmentation models.

Based on CellBinDB, we evaluated the performance of cell segmentation models on a series of benchmark tasks. CellBinDB is entirely new and unseen for all the evaluated models. We first evaluated the robustness and generalization of the cell segmentation models on the entire dataset. Our results show that without retraining and fine-tuning, the non-deep learning method CellProfiler has limited generalization ability and its performance significantly lags behind deep learning models. In stark contrast, deep learning models, especially Cellpose3, show excellent segmentation and generalization performance, highlighting their superiority in this field. Considering the significant difference in different staining types and image features learned by various models from training set, we then evaluated the cell segmentation models on image subsets of four staining types separately. In this evaluation, we found the challenges of cell segmentation on H&E-stained images and mIF-stained images and subsequently proposed a corresponding solution. Based on the above cell segmentation tasks, we conducted a comprehensive evaluation of the model performance and provided model selection suggestions for different cell segmentation scenarios.

Subsequently, we explore the factors that affect cell segmentation results. First, from the biological factors of the samples, differences in cell morphology such as cell area, cell density, and roundness directly affect the difficulty of cell segmentation. We used four indicators that were positively

correlated with F1 score to quantify this effect. Different tissue types are directly related to cell morphology. We provide the normalized scores of cell morphological indicators for each tissue in CellBinDB to quantify the difficulty of cell segmentation in different tissues. Through this evaluation, we can estimate the performance of the model by the tissue type of the segmented image. For example, we can predict that cell segmentation in tissues such as the small intestine, ganglion, and tongue will be challenging. Finally, the evaluation of the impact on image quality shows that high image gradient magnitude facilities cell segmentation. The error factors such as image noise, blur, etc. will affect image gradient, which is possibly due to the limitations of the optical system itself, the sample preparation process, and the uncertainty of imaging conditions. Therefore, laboratory personnel should strictly perform experimental operations to provide higher quality image data for downstream systems.

We fine-tuned the models participating in the benchmark on CellBinDB and subsequently assessed their performance on independent third-party datasets containing previously unseen images. The fine-tuned models show performance improvements, validating CellBinDB's efficacy as a valuable resource for advancing cell segmentation research.

Acknowledging the substantial challenges associated with cellular image data acquisition in this domain, we have made available the 9 public datasets referenced in this study, maintaining their original formats to ensure compatibility and facilitate future research endeavors.

While this work has made a contribution, it is important to acknowledge its limitations. The dataset utilized in this study is confined to two-dimensional (2D) images despite the variety of images. In recent years, the popularity of three-dimensional (3D) images has increased, posing new and unique challenges for cell segmentation tasks. To address these emerging challenges, researchers have already developed algorithms capable of performing 3D cell segmentation. Acknowledging the importance of this advancement, we intend to delve into this direction and explore the potential of incorporating 3D images into CellBinDB in future research endeavors.

In conclusion, compared to any previous work, this work makes a contribution in diversity and scale of dataset, offering a comprehensive and extensive dataset in this work that serves as a valuable resource for advancing cell segmentation models. Nevertheless, this represents only a small step forward in the direction of universal model development. Given the complexities and nuances in the field, ongoing research and continuous expansion of datasets encompassing various staining types, tissue types, and imaging modalities are necessary. By continuing to build upon this preliminary progress, we can collectively work towards the ultimate goal of developing models that can generalize and adapt to diverse datasets, staining techniques, and tissue types in the field of cell segmentation.

Methods

The code library of this study is implemented in Python3[33], using numpy[34], scipy[35], pandas[36], skimage[37], opencv[38] and sklearn[29], and jupyter[39] was used to create a notebook version. The figures

in this article were plotted using matplotlib[40] and seaborn[41], and formatted using Adobe Illustrator.

Data collection and annotation

Image acquisition

CellBinDB includes 29 mouse tissues, 1 rat tissues and 5 human tissues. The 5 different human tissue samples are from the open platform 10x Genomics, with download links provided in the Data Availability section. For two IF-stained images (human skin melanoma and human prostate cancer), only the DAPI channel was selected. The tissues used in this study were provided by the Guangzhou Institutes of Biomedicine and Health, Chinese Academy of Sciences and the BGI laboratory. Mouse tissues were obtained from 6-week-old C57BL/6 female and male experimental mice. Rat tissues were obtained from 10-week-old male SD (Sprague-Dawley) rats. All experimental protocols for generating dataset adhered to ethical regulations regarding animal research. Fresh frozen samples were prepared and sectioned according to STOmics Stereo-seq Transcriptomics Set User Manual. Tissue slices of 10 μm were attached to the chip surface and stained after the tissue fixation. During the imaging stage, the epi-bright field (color camera) mode was selected for H&E-stained tissues while the epi-fluorescence mode was selected for fluorescent-stained tissues. After completing the photography according to the manual requirements, the imageQC module of the StereoMap software was used for image quality control.

WSIs are generated by 1. a STOmics Microscope Go Optical equipped with Scanner Version 1.2.2, using 10×/0.75 NA and 20X/0.5 NA objective and Go Optical Scanner Ximea Mc124 for grayscale images and Go Optical Scanner Ximea Mc050 for RGB images. 2. a Motic PA53 FS6 Microscope equipped with PA53Scanner 1.0.0.14, using 10×/0.75 NA objective and PA53 FS6 SCAN S5LITE MONO for grayscale images and PA53 FS6 SCAN S5LITE for RGB images. We obtained whole slide images (WSIs) from the laboratory or open platforms and then cropped the size to 512×512-pixel or 256×256-pixel sizes. A biologist selected the most representative fields of view (FOV) for each WSI, ensuring that the selected FOV images were clear and suitable for creating ground truth.

Image annotation

We trained a model on CellBinDB and trained a team of professional annotators to semi-automatically annotate the dataset proposed in this study through three steps: model segmentation, manual modification or re-annotation, and expert verification. The model we used for annotation was from Li, M. *et al.*[42] The model architecture was psaUnet contained 5 encoder blocks and 5 decoders blocks. In the preprocessing stage, median filtering was used to smooth the possible noise in the input image. And in the post-processing stage, operations such as corrosion, dilation, and watershed were performed. The preliminary results of the model segmentation were saved in Tagged Image File Format (TIFF) and then converted to JavaScript Object Notation (json) format and imported into Qupath 0.4.3 software[43]. In Qupath 0.4.3, the nuclei annotated by the model were visualized as polygons overlaid on the original nucleus images. If the model segmentation results were satisfactory, the annotation team obtained more accurate annotations by refining polygon

outlines, deleting or adding polygons to modify the results of the model segmentation. Conversely, the annotation team re-annotated manually. The model we trained did not support cell membrane segmentation, therefore mIF-stained images must be annotated manually. Meanwhile the model annotation step was skipped, and the annotation team directly used Qupath 0.4.3 software to outline the cells with the polygon tool. These annotations were subsequently checked by experts. The incorrectly annotated images and corresponding modification suggestions were returned to the annotation team. Once the annotations were approved, the two types of masks: instance mask and semantic mask, were exported and converted into TIFF. Finally, the microscope images were added to the dataset along with the two forms of masks. Supplementary Table 2 provides detailed information such as the file name and staining type, tissue type, size, and data source for each image.

The annotation of cell is challenging due to out-of-focus or nuclei presenting with modified morphology during the slide preparation procedure. We defined the following criteria to annotate images:

- Naked eye is the highest standard and all cells that can be identified by naked eye are annotated ensuring no false negatives (FN) or false positives (FP).
- The entire cell must be completely covered without any omissions or obvious gaps.
- The annotated cell boundaries should be smooth without excessive jagged edges.
- Overlapping cells should be annotated separately.
- The segmentation should not be excessively fragmented. For elongated cells, do not split them into multiple individuals.

Model architecture and default parameters

SAM[25] claims that it has capability on Zero-shot generalization of new image distributions and tasks. The model_type of SAM utilizes ‘vit_b’ and applies SamAutomaticMaskGenerator to automatically generate masks without the need for external prompts.

Cellpose1[5] can segment multiple types of cells without requiring parameter adjustments, new training data or further model retraining. Cellpose1 uses the ‘cyto’ model, with the channels for grayscale images set to [0,0] and [1,3] for H&E images. The diameter is set to None.

Cellpose3[6] introduced a novel method for biological image that achieves efficient segmentation without the requirement for clean images. Cellpose3 uses the ‘cyto3’ model, with the channels for grayscale images set to [0,0] and [1,3] for H&E images. The diameter is set to None, and the ‘denoise_cyto3’ model is used for noisy images.

DeepCell[7] achieves human-level accuracy across a variety of tissues and imaging modalities while requiring no manual parameter tuning for the end user. DeepCell uses the ‘Mesmer’ model, with image_map set to 0.5 and compartment set to ‘nuclear’.

MEDIAR[26] stood out in the NeurIPS 2022 cell segmentation competition[24] and achieved state of the art (SOTA). MEDIAR uses the provided from_phase2.pth model, with model_args configured as follows: ‘classes’ is set to 3, ‘decoder_channels’ is set to [1024, 512, 256, 128, 64], ‘decoder_pab_channels’ is set to 256, ‘encoder_name’ is set to ‘mit_b5’, and ‘in_channels’ is set to

3. The algo_params has ‘use_tta’ set to True.

StarDist[9] uses star-convex polygons to represent cell shapes, allowing accurate cell localization even under challenging conditions. StarDist uses the ‘2D_versatile_he’ model for HE images and the ‘2D_demo’ model for non-HE images.

Cellprofiler[8] is one of the earliest high-throughput cell image analysis platforms. Cellprofiler segmentation pipeline for non-H&E images is as follows: firstly, use IdentifyPrimaryObjects to identify cell objects, setting “Discard objects touching the border of the image” to No. Then, apply OverlayOutlines to outline the cells. Next, use ExpandOrShrinkObject to erode the cell boundaries, separating closely adjacent cells. MaskImage is utilized to convert the image to a mask and finally, save the image with SaveImage. For H&E images, the segmentation pipeline is similar but it starts with an additional step: convert the image to grayscale and then invert the colors before proceeding with the same steps as for non-H&E images.

HoVerNet[30] is a novel convolutional neural network that enables simultaneous segmentation and classification of nuclei in histology images spanning multiple tissue types. We employed the default parameters provided by the official implementation, using both the tile and original modes. The settings were as follows: nr_types=0, nr_inference_workers=8, nr_post_proc_workers=16, and batch_size=32. Additionally, we utilized the CPM-17 checkpoint model weights released by the official source.

Fine-tuning pipeline

For Cellpose and Cellpose3, we conducted fine-tuning using the cyto and cyto3 pretrained weights, respectively, in accordance with the official Cellpose 2 documentation. The fine-tuning process employed default hyperparameters and was performed on the complete set of images from the CellbinDB dataset.

For MEDIAR, we utilized the finetuning1.json configuration file provided in the official fine-tuning procedure, similarly using the entire CellbinDB dataset.

Following fine-tuning, we evaluated all three models on the HE-stained dataset IEEE_TMI_2019. The results indicated that the fine-tuning process enhanced the HE segmentation performance of all three models.

Benchmark pipeline

Different from some previous work[27,44–46], we designed a series of benchmarks to test the performance of each model without retraining. The method maximized the diversity and breadth of CellBinDB to evaluate the generalizability and robustness of each model. Unless otherwise specified, default parameters will be used. Each step of the benchmark targets a different cell segmentation scenario:

(1) Model evaluation on the entire dataset. First, we test each model on the entire dataset including all staining types and tissue types and evaluate the overall performance results using the

metrics precision, recall, F1 score, and Dice. The step aims to recommend the model with the optimum overall performance when the user is segmenting a multimodal dataset or user id unfamiliar with their own data's attributes.

(2) Model evaluation on different staining types. In this step, the dataset is classified into four staining types and the performance of each model is tested individually on each single staining type. This step is intended to furnish users with model recommendations for cell segmentation reliant on specific stain types, as well as to facilitate a comparison of the relative challenges imposed by different staining types.

(3) Evaluating the impact of cell morphology on the performance of cell segmentation models. To quantify cell morphology, we measured a series of metrics for each cell in the dataset, which represent different cell characteristics. We then selected several metrics (cell area, average distance, cell circularity, and cell compactness) with the greatest impact on the F1 score to explore the relationship between cell morphology and model performance. Initially, the values of these four indicators were computed for each cell, and the mean value for each image was determined. Subsequently, the images were categorized into three groups—low, medium, and high—based on tertiles, to assess whether there are significant differences in the F1 scores across these groups. Given the similarity in features between DAPI and ssDNA-stained images, these two types were analyzed together. In contrast, H&E-stained images were analyzed separately, and mIF-stained images were excluded from this experiment due to their unique membrane staining characteristics.

(4) Exploring the effect of cellular image gradients on model segmentation performance. First, we matched individual cells with ground truth and predicted results. Then, we calculated the gradient magnitude of each cell using the Sobel operator in OpenCV, along with its corresponding F1 score. Subsequently, cells were categorized into three groups—low, medium, and high gradients—to assess whether there were significant differences in F1 scores across these groups.

Metrics

Segmentation benchmark

To evaluate models' performance on benchmark pipeline, an evaluation protocol was used which is calculated in several steps. First, the overlap between each prediction and its closest ground truth object is quantified using the *IoU* as follows:

$$IoU = \frac{P \cap G}{P \cup G}$$

where *P* is prediction, *G* is ground truth. If the *IoU* between the prediction and the closest ground truth object is greater than 0.5, the ground truth object is considered to be correctly segmented. For all ground truth objects, the segmentation performance is then quantified using the *precision* and *recall* metrics given below:

$$precision = \frac{TP}{TP + FP}$$

$$recall = \frac{TP}{TP + FN}$$

where *TP* is the number of true positives, *FP* is the number of false positives and *FN* is the number of false negatives. The *F*1 *score* is a metric used to assess the balance between *precision* and *recall*. It is the harmonic mean of *precision* and *recall*, providing a single score that encapsulate the characteristics of both parameters:

$$F1\ score = 2 \times \frac{precision \times recall}{precision + recall}$$

In addition, a pixel-level evaluation metrics is introduced:

$$dice = \frac{2\,|A \cap B|}{|A| + |B|} = \frac{2 \times TP}{2 \times TP + FP + FN}$$

We propose to also use another metric that can be accurately quantified and interpreted to evaluate the performance of kernel instance segmentation, the panoptic quality (PQ) of kernel instance segmentation[47], defined as:

$$PQ = \frac{|TP|}{|TP| + \frac{1}{2}|FP| + \frac{1}{2}|FN|} \times \frac{1}{|TP|} \sum_{(x,y)\in TP} IoU(x, y)$$

Cytomorphological indicators

To explore the impact of cell morphology on segmentation performance, we measured a series of morphological metrics for each cell in the dataset and selected the ones with the greatest impact, including cell area, average cell distance, cell circularity, and cell compactness.

The cell area is the number of pixels contained in each cell. The average cell distance refers to the average distance between each cell and the center point of the nearest cell. Cell circularity is used to calculate the circularity of the cell shape. The closer the value is to 1, the closer the cell shape is to a circle. We used the cv2.convexHull() function in OpenCV to get the convex hull of each cell, used the cv2.arclength function to get the convex hull area, and then calculated the circularity according to the following formula:

$$Circularity = \frac{4\pi \times Area}{Convex.P^2}$$

Compactness is the degree of compactness of the cell shape, calculated based on the cell area and cell perimeter. The closer the compactness is to 1, the more compact and circular the cell shape is; the closer the compactness is to 0, the more irregular and scattered the cell shape is:

$$Compactness = \frac{4\pi \times Area}{P^2}$$

The difference between the above two indicators is that cell roundness uses the perimeter of the cell convex hull, while compactness uses the cell perimeter. If there are many irregular protrusions or depressions on the edge of the cell, the value of compactness will obviously deviate from 1. Circularity pays more attention to the roundness of the overall shape and is less affected by slight irregularities on the edge.

In order to more intuitively display the association between cell morphology indicators and tissue

types, the above four morphology indicators need to be normalized before drawing the heat map, so that indicators of different units or magnitudes can be compared and weighted. The commonly used method is linear transformation, which is to scale the data to a specific range [0, 1]. The calculation formula is as follows:

$$x' = \frac{x - x_{min}}{x_{max} - x_{min}}$$

Where x is the original value. x_{min} and x_{max} are the minimum and maximum values of the metric in the entire dataset, respectively, x' is the normalized value.

Image gradient calculation

In image processing, gradient magnitude is an important indicator to measure the rate of change of pixel intensity in an image. It plays a key role in tasks such as edge detection. To calculate the gradient magnitude, we use the Sobel operator, which is one of the most important operators for image edge detection. The Sobel operator approximates the gradient values of the image in the horizontal(G_x) and vertical(G_y) directions through two 3x3 convolution kernels. These two convolution kernels perform convolution operations on the image separately to highlight the changes along their respective axes. Specifically, the Sobel operator uses the weighted difference of neighboring pixels to emphasize these changes.

We used the ground truth mask to identify the cell regions in the cell images, then applied the Sobel operator from OpenCV (cv2) to compute the x and y gradients of the cell images, followed by calculating the gradient magnitude and average.

The formula for the gradient in the x-direction (G_x):

$$G_x = I * S_x$$

where *I* is the input image, and S_x is the Sobel kernel matrix for the x-direction:

$$S_x = \begin{bmatrix} -1 & 0 & 1 \\ -2 & 0 & 2 \\ -1 & 0 & 1 \end{bmatrix}$$

The formula for the gradient in the y-direction (G_y):

$$G_y = I * S_y$$

where S_y is the Sobel kernel matrix for the y-direction:

$$S_y = \begin{bmatrix} -1 & -2 & -1 \\ 0 & 0 & 0 \\ 1 & 2 & 1 \end{bmatrix}$$

The gradient magnitude (G) is calculated as:

$$G = \sqrt{G_x^2 + G_y^2}$$

It combines the gradient information in two directions and provides a scalar value that is proportional to the edge strength of each point in the image. In short, higher gradient magnitude values usually indicate significant edge locations in the image, which is crucial for cell segmentation tasks.

The average gradient magnitude is calculated as:

$$Average\ Gradient\ Magnitude = \frac{1}{N}\sum_{i=1}^{N} G_i$$

where N is the number of pixels in the image, and G_i is the gradient magnitude of each pixel.

Simulation experiments

We used several different Gaussian blur parameters and adjusted the gradients of these blurred images to create three categories: low, medium, and high gradients. Each category of images had varying levels of blur, aimed at simulating images with different gradient magnitudes in order to assess the impact of gradient on cell segmentation performance. The blurring was implemented using OpenCV's GaussianBlur function. The parameters for low-intensity blurring were a (5,5) kernel size with sigmaX set to 1; for medium intensity, a (9,9) kernel size with sigmaX set to 2; and for high intensity, a (13,13) kernel size with sigmaX set to 3. After applying the blur, we calculated the average gradient magnitude of the images. The calculation formula for Gaussian blurring is as follows:

$$Iblur(x,y) = i = -k\sum^{k} j = -k\sum^{k} I(x+i, y+j) \cdot G(i,j)$$

Where I(x+i,y+j) is the original pixel intensity at position (x+i,y+j), *G(i,j)*is the Gaussian kernel weight for the position *(i,j)* relative to the center.

Data availability

CellBinDB has been deposited into CNGB Sequence Archive (CNSA) of China National GeneBank DataBase (CNGBdb) with accession number CNP0006370: https://db.cngb.org/search/project/CNP0006370/ . We have also uploaded CellBinDB and the 9 public datasets mentioned in Figure 1e to Zenodo[48]. In addition, we uploaded 844 images of mouse and rat from CellBinDB to BioImage Archive[49]. All additional supporting data are available in the GigaScience repository, GigaDB[50]. DOME-ML annotations are available in DOME registry[51].

The 9 public datasets are publicly available[52–60].

The raw data of 5 human samples from 10x Genomics are available free of charge. The download links are as follows:

H&E human lung cancer: https://www.10xgenomics.com/datasets/preview-data-ffpe-human-lung-cancer-with-xenium-multimodal-cell-segmentation-1-standard.

H&E human pancreas: https://www.10xgenomics.com/datasets/ffpe-human-pancreas-with-xenium-multimodal-cell-segmentation-1-standard.

H&E human ovarian cancer: https://www.10xgenomics.com/datasets/ffpe-human-ovarian-cancer-data-with-human-immuno-oncology-profiling-panel-and-custom-add-on-1-standard.

DAPI human skin melanoma: https://www.10xgenomics.com/datasets/human-melanoma-if-stained-ffpe-2-standard .

Human DAPI human prostate Cancer: https://www.10xgenomics.com/datasets/human-prostate-cancer-adjacent-normal-section-with-if-staining-ffpe-1-standard .

Code availability

Github project home page: https://github.com/STOmics/cs-benchmark.

Programming language: Python

Other requirements: Python 3.8 or higher

License: MIT License

RRID: SCR_026740

BiotoolsID: cellbindb

Software Heritage PID[61]:
https://archive.softwareheritage.org/swh:1:snp:4248eacf2a6512e456b43bb9c9adc8f140d41051.

Abbreviations

DAPI: 4',6-Diamidino-2-Phenylindole

ssDNA: Single-stranded DNA

H&E: Hematoxylin and Eosin

mIF: Multiplex Immunofluorescence

Authors' Contributions

Project administration and supervision: Mei Li, Ying Zhang

Algorithm development and implementation: Jinghong Fan, Huanlin Liu, Zhonghan Deng

Data provided: Yumei Li, Jing Guo

Data selection, processing and sorting: Can Shi

Data annotation: Can Shi, Jinjiang Gong, Jingwen Wang, Jinghong Fan, Ying Zhang, Yumei Li

Project coordination: Mei Li, Ying Zhang, Zhonghan Deng

Method comparisons: Can Shi, Jinghong Fan

Code Testing: Can Shi, Jinghong Fan

Manuscript writing and figure generation: Can Shi, Jinghong Fan, Ying Zhang

Manuscript review: Can Shi, Jinghong Fan, Mei Li, Ying Zhang, Qiang Kang, Sha Liao, Ao Chen

Competing interests

The authors declare they have no competing interests.

Acknowledgments

We thank the China National GeneBank for providing data storage support for this study, and the National Key R&D Program of China (2022YFC3400400) for funding. We would also like to express our gratitude to Dr. Shuangsang Fang, Dr. Min Xie, Tongxuan Lv, Ning Feng, Zepeng Li, Jiao Qiao, Manqi Liang, Jiaxue Chen, Huiying Du, and Qiushuo Wang for their assistance.

Reference

1. Hartmann, R. *et al.* Quantitative image analysis of microbial communities with BiofilmQ. *Nat Microbiol* **6**, 151–156 (2021).
2. Sigal, Y. M., Zhou, R. & Zhuang, X. Visualizing and discovering cellular structures with super-resolution microscopy. *Science* **361**, 880–887 (2018).
3. Cui, M. & Zhang, D. Y. Artificial intelligence and computational pathology. *Lab Invest* **101**, 412–422 (2021).
4. Ronneberger, O., Fischer, P. & Brox, T. U-Net: Convolutional Networks for Biomedical Image Segmentation. in *Medical Image Computing and Computer-Assisted Intervention – MICCAI 2015* (eds. Navab, N., Hornegger, J., Wells, W. M. & Frangi, A. F.) vol. 9351 234–241 (Springer International Publishing, Cham, 2015).
5. Stringer, C., Wang, T., Michaelos, M. & Pachitariu, M. Cellpose: a generalist algorithm for cellular segmentation. *Nat Methods* **18**, 100–106 (2021).
6. Stringer, C. & Pachitariu, M. Cellpose3: one-click image restoration for improved cellular segmentation. Nat Methods 22, 592–599 (2025).
7. Greenwald, N. F. *et al.* Whole-cell segmentation of tissue images with human-level performance using large-scale data annotation and deep learning. *Nat Biotechnol* **40**, 555–565 (2022).
8. Carpenter, A. E. *et al.* CellProfiler: image analysis software for identifying and quantifying cell phenotypes. *Genome Biol* **7**, R100 (2006).
9. Schmidt, U., Weigert, M., Broaddus, C. & Myers, G. Cell Detection with Star-Convex Polygons. in *Medical Image Computing and Computer Assisted Intervention – MICCAI 2018* (eds. Frangi, A. F., Schnabel, J. A., Davatzikos, C., Alberola-López, C. & Fichtinger, G.) vol. 11071 265–273 (Springer International Publishing, Cham, 2018).
10. He, K., Gkioxari, G., Dollar, P. & Girshick, R. Mask R-CNN. IEEE Trans. Pattern Anal. Mach. Intell. 42, 386–397 (2020).
11. Lee, Y. & Park, J. CenterMask: Real-Time Anchor-Free Instance Segmentation. in 2020 IEEE/CVF Conference on Computer Vision and Pattern Recognition (CVPR) 13903–13912 (IEEE, Seattle, WA, USA, 2020). doi:10.1109/CVPR42600.2020.01392.
12. Cutler, K. J. *et al.* Omnipose: a high-precision morphology-independent solution for bacterial cell segmentation. *Nat Methods* **19**, 1438–1448 (2022).
13. Xie, E. et al. SegFormer: Simple and Efficient Design for Semantic Segmentation with Transformers. in Advances in Neural Information Processing Systems (eds. Ranzato, M., Beygelzimer, A., Dauphin, Y., Liang, P. S. & Vaughan, J. W.) vol. 34 12077–12090 (Curran Associates, Inc., 2021).

14. Kang, M., Ting, C.-M., Ting, F. F. & Phan, R. C.-W. ASF-YOLO: A Novel YOLO Model with Attentional Scale Sequence Fusion for Cell Instance Segmentation. Preprint at https://doi.org/10.1016/j.imavis.2024.105057 (2024).
15. Kumar, N. *et al.* A Multi-Organ Nucleus Segmentation Challenge. *IEEE Trans. Med. Imaging* **39**, 1380–1391 (2020).
16. Ma, J. & Wang, B. Towards foundation models of biological image segmentation. *Nat Methods* **20**, 953–955 (2023).
17. Tizhoosh, H. R. & Pantanowitz, L. Artificial Intelligence and Digital Pathology: Challenges and Opportunities. *J Pathol Inform* **9**, 38 (2018).
18. Caicedo, J. C. *et al.* Nucleus segmentation across imaging experiments: the 2018 Data Science Bowl. *Nat Methods* **16**, 1247–1253 (2019).
19. Naylor, P., Laé, M., Reyal, F. & Walter, T. Segmentation of Nuclei in Histopathology Images by Deep Regression of the Distance Map. *IEEE Trans. Med. Imaging* **38**, 448–459 (2019).
20. Kromp, F. *et al.* An annotated fluorescence image dataset for training nuclear segmentation methods. *Sci Data* **7**, 262 (2020).
21. Graham, S. *et al.* Lizard: A Large-Scale Dataset for Colonic Nuclear Instance Segmentation and Classification. in *2021 IEEE/CVF International Conference on Computer Vision Workshops (ICCVW)* 684–693 (2021). doi:10.1109/ICCVW54120.2021.00082.
22. Mahbod, A. *et al.* NuInsSeg: A fully annotated dataset for nuclei instance segmentation in H&E-stained histological images. *Sci Data* **11**, 295 (2024).
23. Caicedo, J. C. *et al.* Evaluation of Deep Learning Strategies for Nucleus Segmentation in Fluorescence Images. *Cytometry Pt A* **95**, 952–965 (2019).
24. Ma, J. *et al.* The multimodality cell segmentation challenge: toward universal solutions. *Nat Methods* **21**, 1103–1113 (2024).
25. Kirillov, A. *et al.* Segment Anything. in *Proceedings of the IEEE/CVF International Conference on Computer Vision (ICCV)* 4015–4026 (2023).
26. Lee, G., Kim, S., Kim, J. & Yun, S.-Young. MEDIAR: Harmony of Data-Centric and Model-Centric for Multi-Modality Microscopy. in *Proceedings of The Cell Segmentation Challenge in Multi-modality High-Resolution Microscopy Images* (eds. Ma, J. et al.) vol. 212 1–16 (PMLR, 2023).
27. Kar, A. *et al.* Benchmarking of deep learning algorithms for 3D instance segmentation of confocal image datasets. *PLoS Comput Biol* **18**, e1009879 (2022).
28. Maaten, L. V. der & Hinton, G. Visualizing data using t-SNE. *Journal of machine learning research* **9**, 2579–2605 (2008).
29. Pedregosa, F., Varoquaux, G., Gramfort, A. & Michel, V. Scikit-learn: Machine learning in Python. *the Journal of machine Learning research* **12**, 2825–2830 (2011).
30. Graham, S. *et al.* Hover-Net: Simultaneous segmentation and classification of nuclei in multi-tissue histology images. *Medical Image Analysis* **58**, 101563 (2019).
31. Bancher, B., Mahbod, A., Ellinger, I., Ecker, R. & Dorffner, G. Improving Mask R-CNN for Nuclei Instance Segmentation in Hematoxylin & Eosin-Stained Histological Images. *MICCAI workshop on computational pathology* 20--35 (2021).
32. Fu, X. *et al.* BIDCell: Biologically-informed self-supervised learning for segmentation of subcellular spatial transcriptomics data. *Nat Commun* **15**, 509 (2024).
33. Rossum, G. van & Drake, F. L. *The Python Language Reference*. (Python Software Foundation, Hampton, NH, 2010).

34. Harris, C. R. *et al.* Array programming with NumPy. *Nature* **585**, 357–362 (2020).
35. Virtanen, P. *et al.* SciPy 1.0: fundamental algorithms for scientific computing in Python. *Nat Methods* **17**, 261–272 (2020).
36. McKinney, W. Data Structures for Statistical Computing in Python. in 56–61 (Austin, Texas, 2010). doi:10.25080/Majora-92bf1922-00a.
37. Van Der Walt, S. *et al.* scikit-image: image processing in Python. *PeerJ* **2**, e453 (2014).
38. Bradski, G. The Opencv Library. *Dr. Dobb's Journal: Software Tools for the Professional Programmer* **25**, 120–125 (2000).
39. Kluyver, T. *et al.* Jupyter Notebooks – a publishing format for reproducible computational workflows. in *Positioning and Power in Academic Publishing: Players, Agents and Agendas* 87–90 (IOS Press, 2016). doi:10.3233/978-1-61499-649-1-87.
40. Hunter, J. D. Matplotlib: A 2D Graphics Environment. *Comput. Sci. Eng.* **9**, 90–95 (2007).
41. Waskom, M. seaborn: statistical data visualization. *JOSS* **6**, 3021 (2021).
42. Li, M. *et al.* CellBin: a highly accurate single-cell gene expression processing pipeline for high-resolution spatial transcriptomics. Preprint at https://doi.org/10.1101/2023.02.28.530414 (2023).
43. Bankhead, P. *et al.* QuPath: Open source software for digital pathology image analysis. *Sci Rep* **7**, 16878 (2017).
44. Maška, M. *et al.* A benchmark for comparison of cell tracking algorithms. *Bioinformatics* **30**, 1609–1617 (2014).
45. Maška, M. *et al.* The Cell Tracking Challenge: 10 years of objective benchmarking. *Nat Methods* **20**, 1010–1020 (2023).
46. Zheng, W. *et al.* NIS3D: A Completely Annotated Benchmark for Dense 3D Nuclei Image Segmentation. in *Advances in Neural Information Processing Systems* (eds. Oh, A. et al.) vol. 36 4741–4752 (Curran Associates, Inc., 2023).
47. Kirillov, A., He, K., Girshick, R., Rother, C. & Dollár, P. Panoptic Segmentation. Preprint at https://doi.org/10.48550/arXiv.1801.00868 (2019).
48. Shi C; Fan J; Deng Z; Liu H; Kang Q; Li Y; Guo J; Wang J; Gong J; Liao S; Chen A; Zhang Y; Li M (2025). CellBinDB: A Large-Scale Multimodal Annotated Dataset. Zenodo. https://doi.org/10.5281/zenodo.14312043
49. Shi C; Fan J; Deng Z; Liu H; Kang Q; Li Y; Guo J; Wang J; Gong J; Liao S; Chen A; Zhang Y; Li M (2024). CellBinDB: A Large-Scale Multimodal Annotated Dataset. BioStudies. https://doi.org/10.6019/S-BIAD1538
50. Shi C; Fan J; Deng Z; Liu H; Kang Q; Li Y; Guo J; Wang J; Gong J; Liao S; Chen A; Zhang Y; Li M (2025): Supporting data for "CellBinDB: A Large-Scale Multimodal Annotated Dataset for Cell Segmentation with Benchmarking of Universal Models" GigaScience Database. https://doi.org/10.5524/102713.
51. Shi C, Fan J, Deng Z, Liu H, Kang Q, Li Y, Guo J, Wang J, Gong J, Liao S, Chen A, Zhang Y and Li M. (2025) CellBinDB: A Large-Scale Multimodal Annotated Dataset for Cell Segmentation with Benchmarking of Universal Models. [DOME-ML Annotations]. DOME-ML Registry, https://registry.dome-ml.org/review/etom5gtugj.
52. MoNuSeg. MoNuSeg dataset: Data. https://monuseg.grand-challenge.org/Data/. Accessed 21 May 2024.
53. IEEE_TMI_2019. Dataset for "Automated detection and classification of nuclei in H&E stained histopathological images". https://doi.org/10.5281/zenodo.1174342. Accessed 21 May 2024.

54. Kromp C, et al. Labeled histopathology image dataset for nucleus segmentation and classification. https://www.ebi.ac.uk/biostudies/bioimages/studies/S-BSST265. Accessed 21 May 2024.
55. Aadimator. Lizard dataset. Kaggle. https://www.kaggle.com/datasets/aadimator/lizard-dataset. Accessed 21 May 2024.
56. Cellpose. Cellpose dataset. https://www.cellpose.org/dataset. Accessed 21 May 2024.
57. IpaTeam. NuInsSeg dataset. Kaggle. https://www.kaggle.com/datasets/ipateam/nuinsseg. Accessed 21 May 2024.
58. Broad Bioimage Benchmark Collection. BBBC038v1 dataset. https://bbbc.broadinstitute.org/BBBC038. Accessed 21 May 2024.
59. Broad Bioimage Benchmark Collection. BBBC039 dataset. https://bbbc.broadinstitute.org/BBBC039. Accessed 21 May 2024.
60. TissueNet Consortium. TissueNet dataset. https://doi.org/10.60597/eaqa-k904. Accessed 21 May 2024.
61. Shi C, Fan J, Deng Z, Liu H, Kang Q, Li Y, Guo J, Wang J, Gong J, Liao S, Chen A, Zhang Y and Li M. (2025) CellBinDB: A Large-Scale Multimodal Annotated Dataset for Cell Segmentation with Benchmarking of Universal Models (Version 1). [Computer software]. Software Heritage, https://archive.softwareheritage.org/swh:1:snp:4248eacf2a6512e456b43bb9c9adc8f140d41051.

Figure legends

Figure 1: CellBinDB overview. (a) Distribution of staining types in CellBinDB. (b) Distribution of tissue types in CellBinDB, where tissue types with less than 10 samples are included in other, for details, see supplementary table 1. (c) Examples of CellBinDB images with scale bar and instance ground truth annotations, from left to right columns 1: ssDNA, columns 2: DAPI, columns 3: H&E, columns 4: mIF, column 5: 10x Genomics DAPI, column 6: 10x Genomics H&E. The first row is the original microscope images and the second contains the instance annotation masks. d, Scatter plot of t-SNE demonstrates the diverse spread of data by different staining types and sources. e, Scatter plot of t-SNE demonstrates the diversity of CellBinDB compared to previous datasets. f, The number of manual and semi-automatic annotations in CellBinDB. g, The dataset annotation process includes four steps: 1.model annotation, 2.annotation team modification/re-annotation(depends on the model annotation results), 3.expert review, go to the next step if the annotations are correct, otherwise return to the second step for modification, 4. add the original image and the two masks to the dataset.

Figure 2. Evaluate model performance on the entire dataset and by staining type. (a) Evaluate model performance on the entire dataset, with bar charts of precision, recall, F1 score, dice and PQ. (b) Model performance evaluation results on DAPI-stained images. (c) Model performance evaluation results on ssDNA-stained images. (d) Model performance evaluation results on H&E-stained images. (e) Model performance evaluation results on mIF-stained images. (f-i) AP-IoU curves of the segmentation results of the model on four stains (DAPI, ssDNA, H&E, mIF). The IoU threshold quantifies the match between the predicted mask and the ground truth mask. (j-m)

Examples of segmentation results for each model on four stains (DAPI, ssDNA, H&E, mIF). The ground truth is represented by the red line, while the model prediction is represented by the yellow line.

Figure 3. Model performance after adding preprocessing. (a) Preprocessing of H&E and mIF stained images. H&E-stained images include two steps: grayscale conversion and color inversion, while mIF-stained images only require color inversion. (b) Comparison of F1 scores before and after adding preprocessing for the model with improved performance on H&E-stained images. (c) Comparison of F1 scores before and after adding preprocessing for the model with improved performance on mIF-stained images. (d) Example of segmentation results before and after adding preprocessing on H&E-stained images. (e) Example of segmentation results before and after image preprocessing on mIF stained images.

Figure 4. Evaluation of the impact of cell morphology. (a) For fluorescent stained images (DAPI and ssDNA, Cellpose1 as an example), four indicators (cellArea, averageDistance, cellCircularity, cellCompactness) are used to evaluate the impact of cell morphology on segmentation performance. The vertical axis of the box plot is the F1 score, and the horizontal axis is the sample divided into three parts: low, medium and high according to the tertile of each indicator. And the significance mark line and p-value are added to the box plot. The number of "*" from 1 to 4 represents p-value less than 0.05, 0.01, 0.001 and 0.0001. (b) Same as subfigure a, results on H&E-stained images. (c) Fluorescent staining images display of low, medium and high instances under four indicators. (d) H&E-stained images display of low, medium and high instances under four indicators. (e) Display of normalized scores for the above four metrics by tissue type. (f) According to the mean of the four cell morphology evaluation indicators of each image, it is divided into three groups: low, medium and high. The bar graph shows the relationship with the F1 score.

Figure 5. F1 Score vs Cell Gradient Groups for Different Algorithms. (a) DAPI-stained images, relationship between cell gradient and F1 score. The vertical axis of the box plot is the F1 score, and the horizontal axis is the sample divided into three parts: low, medium and high according to the tertile of cell gradient. And the significance mark line and p-value are added to the box plot. The number of "*" from 1 to 4 represents p-value less than 0.05, 0.01, 0.001 and 0.0001. (b) Same as subfigure a, results on ssDNA-stained images. (c) Same as subfigure a, results on H&E-stained images. (d) Simulation Experiment Results. (e) examples of low, medium and high Gaussian blurred images.

Supplementary Figure1. Performance comparison before and after fine-tuning. (a) Comparison of F1 scores of Cellpose1, Cellpose3 and MEDIAR on the IEEE_TMI_2019 dataset before and after fine-tuning on CellBinDB. (b) Comparison of F1 scores of Cellpose1, Cellpose3 and MEDIAR on the Lizard dataset before and after fine-tuning on CellBinDB. (c) Examples of original images, ground truth, and segmentation results of Cellpose1, Cellpose3, and MEDIAR before and after fine-tuning for the IEEE_TMI_2019 and Lizard datasets using.

Supplementary Figure2. Fluorescent staining and H&E staining images (except for the models shown in Figure 4), four indicators (cellArea, averageDistance, cellCircularity, cellCompactness) are used to evaluate the effect of cell morphology on segmentation performance. The vertical axis

of the box plot is the F1 score, and the horizontal axis is the classification of samples into low, medium, and high parts according to the tertiles of each indicator. Significant marker lines and p-values are added to the box plot. "*" from 1 to 4 indicates that the p-value is less than 0.05, 0.01, 0.001, and 0.0001.

Click here to access/download;Figure;Figure 1.pdf

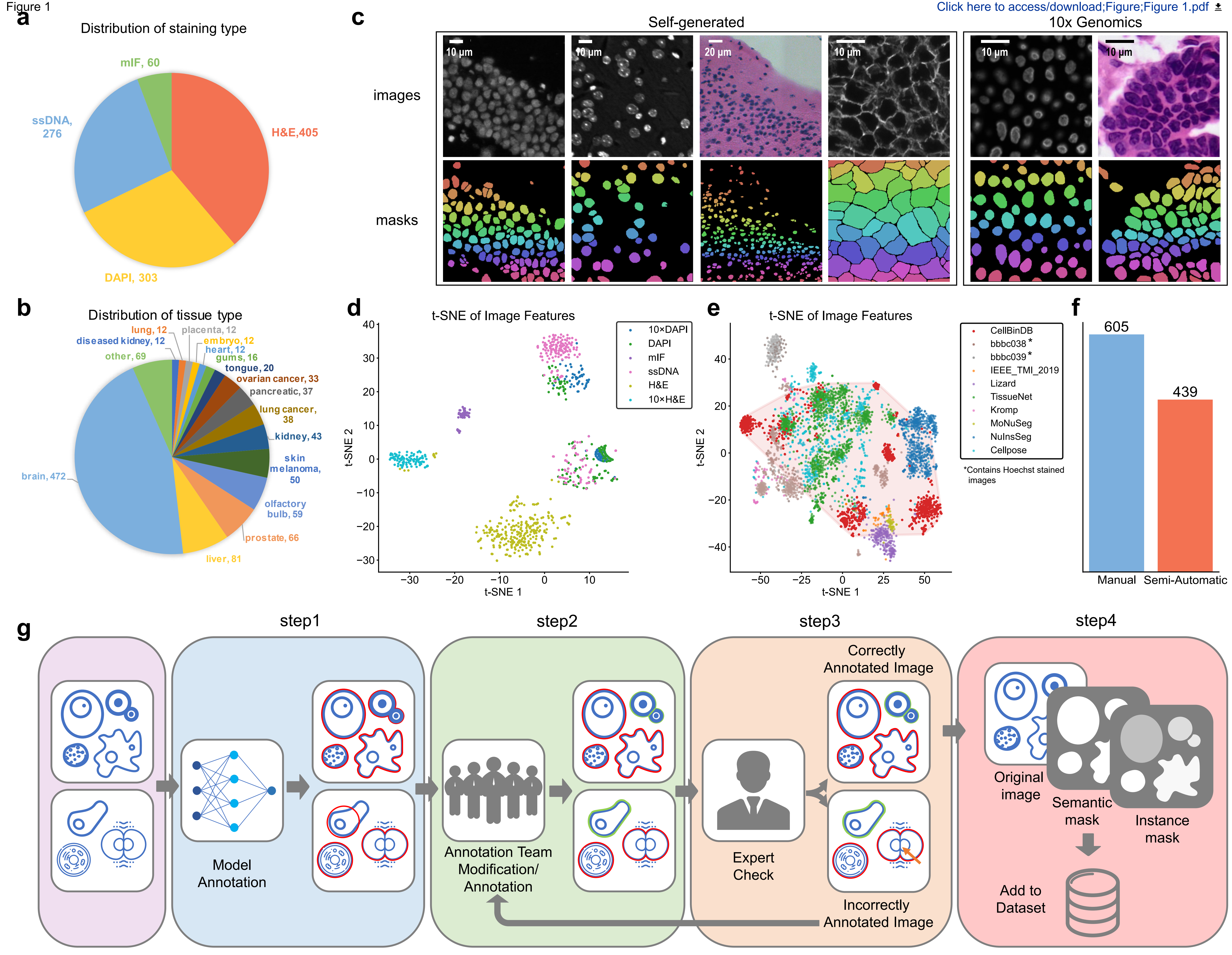

Figure 2 Click here to access/download;Figure;Figure2.pdf

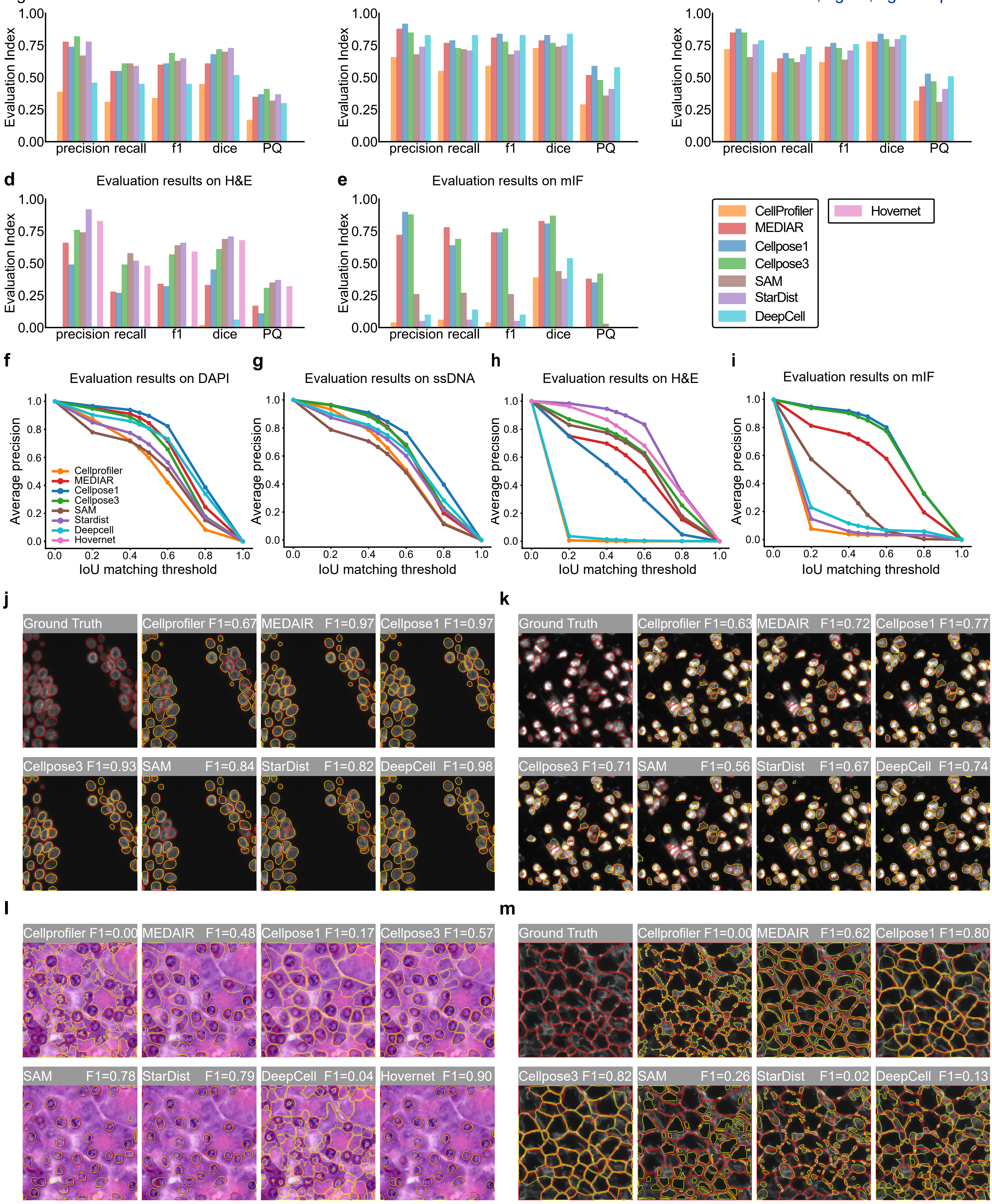

Figure 3 Click here to access/download;Figure;Figure 3.pdf

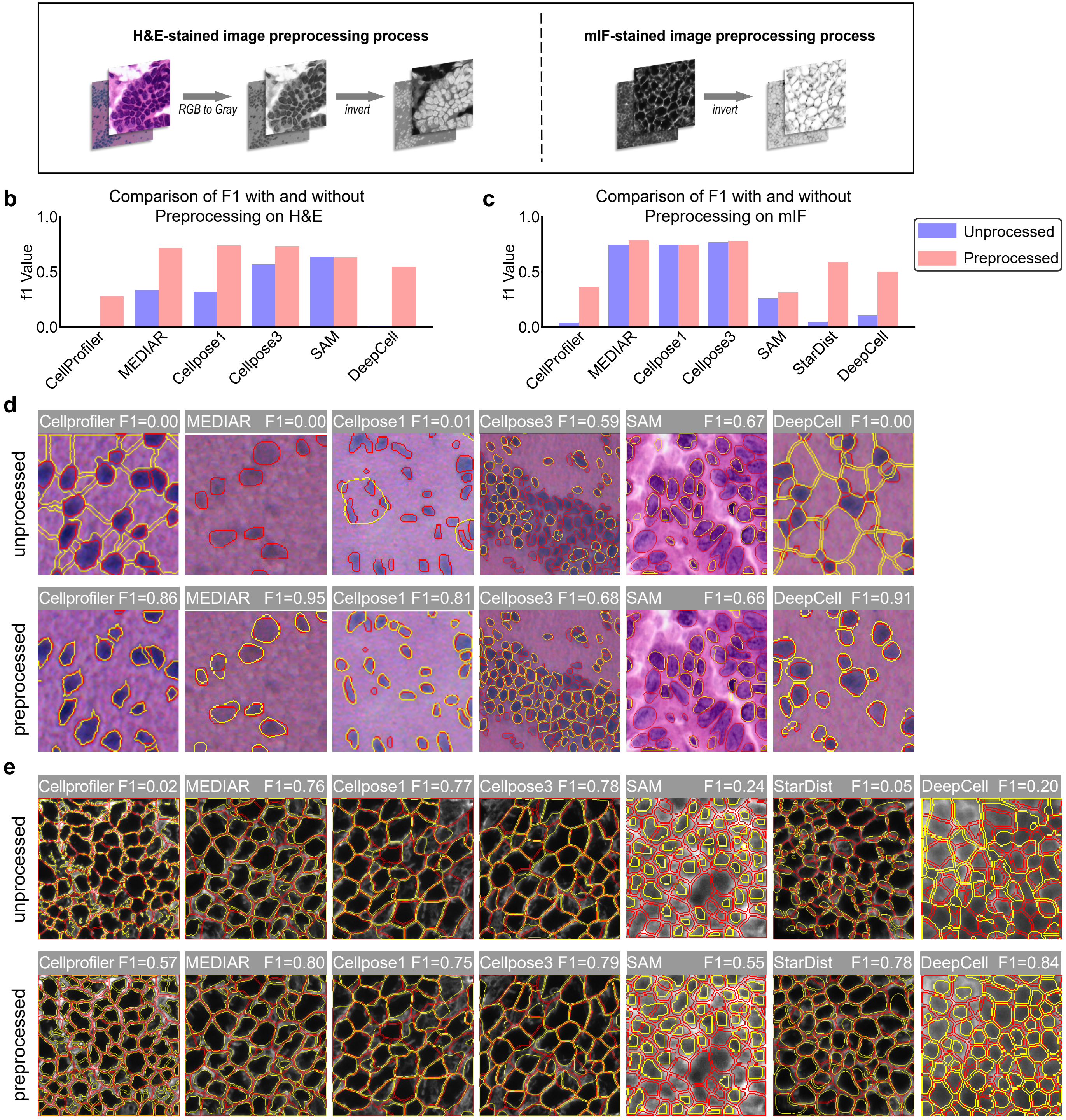

Figure 4

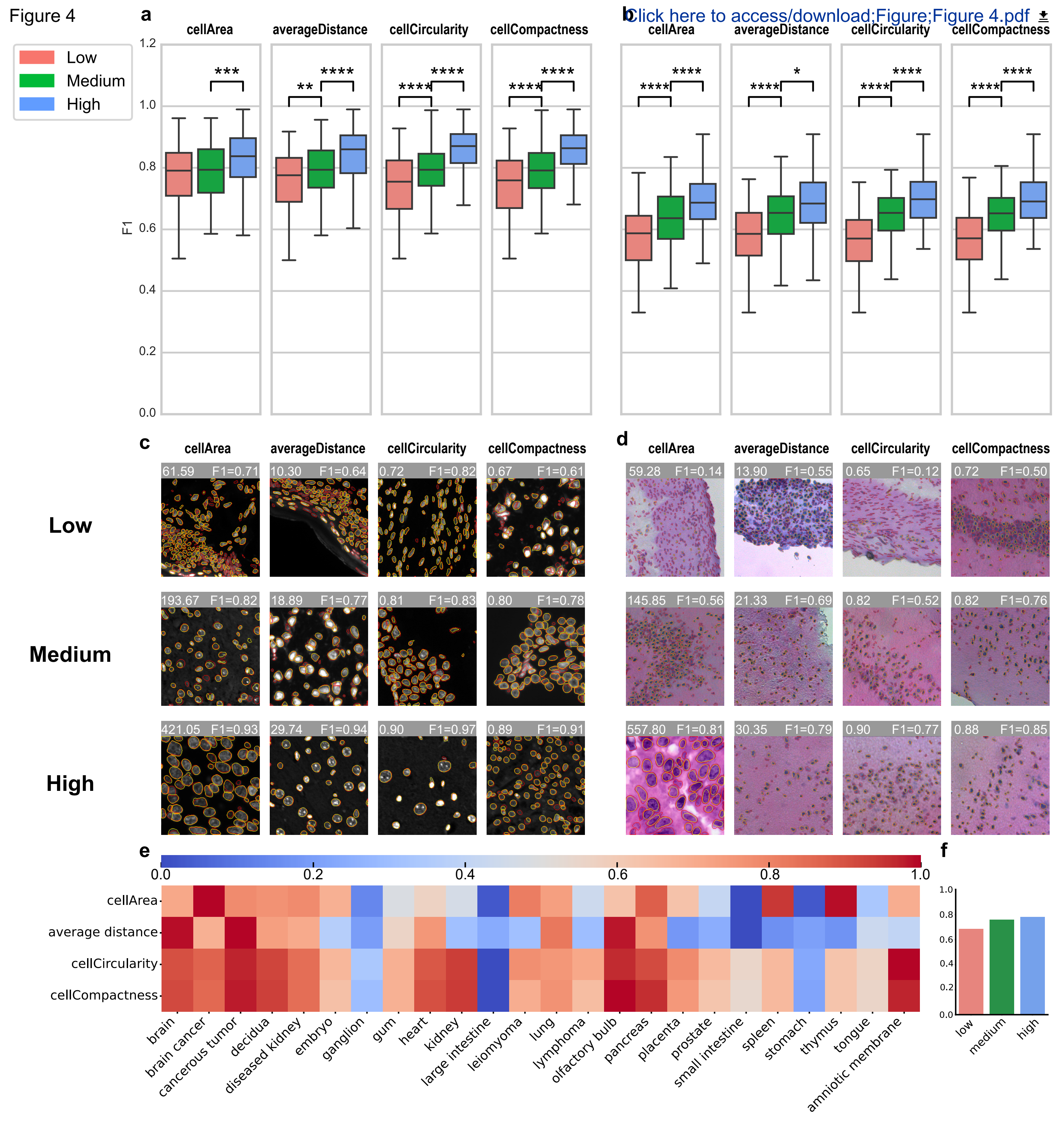

Click here to access/download;Figure;Figure 4.pdf

Figure 5 Click here to access/download;Figure;Figure 5.pdf

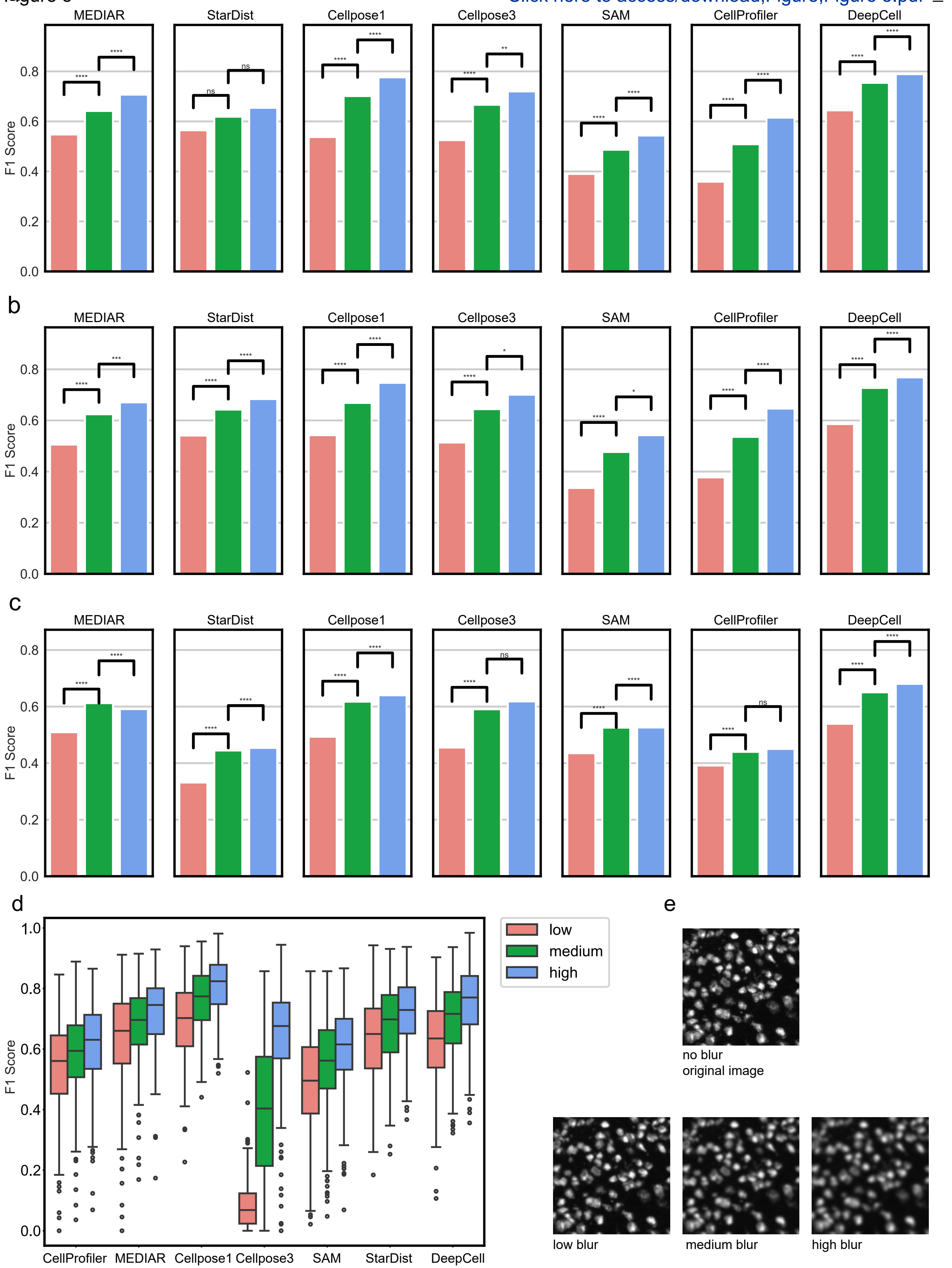

Supplementary Table 1

Click here to access/download
Supplementary Material
Supplementary Table1.xls

Supplementary Table 2

Click here to access/download
Supplementary Material
Supplementary Table2.xls

Supplementary Figure 1

Click here to access/download
Supplementary Material
Supplementary Figure 1.pdf

Supplementary Figure 2

Click here to access/download
Supplementary Material
Supplementary Figure 2.pdf
