# Supplementary material for: CellBinDB: a large-scale multimodal annotated dataset for cell segmentation with benchmarking of universal models
Source: Gigascience. 2025 Jun 24;14:giaf069. doi: 10.1093/gigascience/giaf069 (PMC12206155; doi:10.1093/gigascience/giaf069)
Supplement: giaf069_Authors_Response_To_Reviewer_Comments_original_submission [file giaf069_authors_response_to_reviewer_comments_original_submission.pdf]

Author's Response To Reviewer Comments

Response to Reviewers

Dear editor and reviewers,

Thank you very much for your careful evaluation and valuable feedback on our manuscript, “CellBinDB: A Large-Scale Multimodal Annotated Dataset for Cell Segmentation with Benchmarking of Universal Models” (GIGA-D-24-00566). We sincerely appreciate the constructive comments, which have helped us improve the quality and clarity of the manuscript. We have revised the manuscript accordingly, and the main changes are highlighted in red. Below, we provide a detailed, point-by-point response to each of the reviewers’ comments.

Authors Response to Comments of Editor

Comment: In addition, please register any new software application in the bio.tools and SciCrunch.org databases to receive RRID (Research Resource Identification Initiative ID) and biotoolsID identifiers, and include these in your manuscript. Computational workflows should be registered in workflowhub.eu and the DOIs cited in the relevant places in the manuscript.
Response: Thanks for your comments.
We have registered our software application on both bio.tools and SciCrunch.org, and the corresponding biotoolsID and RRID have been added to the Code availability section of the revised manuscript. Regarding the suggestion to register our computational workflow on workflowhub.eu, we would like to clarify that our computational pipeline was developed entirely using custom Python scripts, and it does not follow a standardized workflow format (e.g., Galaxy, Nextflow, CWL) that is compatible with workflowhub.eu. Therefore, it may not be appropriate to register our code as a workflow in workflowhub.eu. However, to ensure reproducibility and facilitate sharing, we have made all source code publicly available on GitHub. This link is also available in the Code availability section of the manuscript.

Authors Response to Comments of Reviewer 1

Major Areas for Improvement:
Comment 1: Preprocessing Uniformity: Apply preprocessing steps uniformly across all segmentation approaches to ensure fair comparisons and avoid bias.
Response 1: Thanks for your suggestion.
For the mIF-stained images, we applied the preprocessing steps (color inversion) to all methods included in the benchmark. For the H&E-stained images, we applied the preprocessing steps (grayscale conversion and color inversion) to all models except Stardist and HoverNet to align their inputs with the expected data distribution. However, for Stardist and HoverNet, we intentionally avoided these preprocessing steps, as both models were specifically optimized for H&E images, and additional preprocessing could potentially shift the data away from their training distribution. For detailed modifications, see the third paragraph of the Evaluation on four staining types respectively section, and Figure 3b-e.

Comment 2: Inclusion of Cellpose3 Training Dataset: The manuscript should include the dataset used for training Cellpose3 in its comparisons. Cellpose3's superior generalist model performance is emphasized, yet the absence of its training dataset in the comparisons raises questions about robustness of the benchmarking.
Response 2: Thanks for your suggestion, we have added the training set of Cellpose3.
Cellpose3 used nine publicly available datasets to train the “cyto3” model. These include Cellpose's own dataset and 8 other public datasets. Considering that the scope of this study is the microscope images of stained cells, not all the datasets used for Cellpose3 training can be added to this study. The details are as follows:
•Cellpose: Cellpose includes a wide range of images, such as fruits, vegetables, artificial materials, fish scales, reptile scales, starfish, jellyfish, sea urchins, rocks, shells, and some bacterial microscopy images. We partially included this dataset by selecting only the microscopy images related to stained cells. Non-

microscopy images and bacterial samples were excluded to maintain focus.
●Omnipose, YeaZ, DeepBacs, LiveCell: were not included in the study. These datasets primarily feature images of bacteria, yeast, nematodes, plants, or were captured using phase contrast microscopy, which are not aligned with our focus on stained cell microscopy images.
●TissueNet, MoNuSeg, BBBC038, BBBC039: These datasets were previously included in the study because they contained microscopy images of stained cells that were highly consistent with our research goals.
Therefore, we only newly added the Cellpose dataset, which is described in the second paragraph of Introduction section, Table 1, and Figure 1e.

Comment 3: Evidence of Dataset Utility: While the dataset's benchmarking is well-done, the manuscript does not provide evidence that models trained on CellBinDB outperform those trained on other datasets. Addressing this, though potentially out of scope, would strengthen the manuscript's impact.
Response 3: We sincerely appreciate the reviewer’s valuable suggestion regarding the comparative analysis of models trained on CellBinDB versus other datasets. While a direct comparison with models trained on alternative datasets would indeed be insightful, we believe that our current experiments already provide evidence of CellBinDB’s utility in a different but equally meaningful way.
Specifically, we conducted fine-tuning experiments to evaluate whether models pre-trained on CellBinDB can improve performance on unseen datasets (details are provided in the last paragraph of the Introduction, the Fine-tuning pipeline section in Methods, the fourth paragraph of the Discussion, and Supplementary Figure 1). The results demonstrate that models initialized with CellBinDB exhibit enhanced generalization ability, achieving superior performance compared to baseline models trained from scratch. This indicates that CellBinDB serves as a high-quality pre-training resource, facilitating better adaptation to new data distributions—a key aspect of dataset utility in real-world applications.
While we acknowledge that a direct head-to-head comparison with other datasets could further strengthen our claims, such an analysis would require extensive benchmarking across multiple existing datasets, which is beyond the scope of this work focused on establishing and validating CellBinDB as a standardized benchmark. Nevertheless, we fully agree that future studies (including our own) could explore this direction, and we hope the current fine-tuning results—along with the dataset’s diversity, scale, and annotation quality—sufficiently support its value to the community.
Thank you for your constructive feedback, which has helped us better highlight CellBinDB’s practical relevance.

Comment 4: Figure Panels:
* Labeling in figure panels should be clearer to enhance interpretability. For instance, indicate whether the instance or semantic masks are being shown and consider making instance segmentation masks colorful to highlight unique IDs.
* Semantic masks could be omitted if space is constrained, as they are largely redundant with instance masks.
* Ensure figures are spaced more evenly throughout the text, ideally located near their first references, to improve readability.

Response 4: Thank you for pointing out the problem with the figure panel in our article.
We reviewed all figure panels and revised unclear labels. In particular, we removed the semantic masks in Figure 1c because they are redundant with instance masks, and added color coding to instance masks to highlight unique IDs. In addition, we split the original Figure 2 into Figure 2 and Figure 3 to ensure that the figures are more evenly spaced throughout the text.

Comment 5: Abstract Clarity: The abstract should better reflect the intellectual contributions of the analysis of segmentation performance factors (i.e. cell morphology and image gradients).
Response 5: Thank you for your suggestion on the Abstract section. We added relevant descriptions of cell morphology and image gradients in the abstract to make the analysis of factors affecting segmentation results more prominent. The details are at the end of the Abstract.

Comment 6: Normalization Methods: Provide details on how cell morphology indicators are normalized in the methods section to ensure reproducibility and clarity.
Response 6: We have provided detailed information and calculation formulas on how to normalize cell morphology metrics in the Metrics of Methods section.

Comment 7: Explanation of Image Gradient: The discussion of gradient magnitude and its calculation using the Sobel operator requires more accessible language. Not all readers will be familiar with this concept, so additional context is essential.
Response 7: We have added a more detailed introduction to the Sobel operator, combined with the specific calculation formula, hoping to help readers without relevant background to understand. The added content is included in the Cytomorphological Indicators section under Metrics of the Methods.

Comment 8: Tissue Classification: Group related tissues, such as "brain", "half brain" and "cerebellum", under a common "neural tissue" category for easier interpretation and analysis.
Response 8: Thank you for your suggestion. For ease of interpretation and analysis, some tissues can be grouped into one general category. After checking the images in CellBinDB, we believe that the image features of “brain”, “half brain” and “cerebellum” are similar, so we group them into the general category of “brain”. Other tissues, such as “large intestine” and “small intestine”, were not combined because their image features were significantly different. For related content, please see Supplementary Table 1, Supplementary Table 2, Figure 1b and Figure 4e.

Additional Suggestions:
Comment 9:
* Address grammatical errors and improve clarity in some sections, such as the benchmarking pipeline description.
* Replace vague terms like “ML-based” when referring to CellProfiler with specific algorithmic descriptions.
* Including public datasets, such as Cellpose, to create a unified, all-inclusive CellBinDB dataset might significantly enhance the resource's utility for machine learning practitioners.

Response 9: Thank you for your suggestions, which are very meaningful for improving the readability of our article. We have checked and revised the possible grammatical errors and unclear references in the manuscript, and uploaded the 9 public datasets involved in this study to our Zenodo project (https://zenodo.org/records/14312044) for the convenience of readers.

Authors Response to Comments of Reviewer 2

Comment 1: The authors missed one of the biggest data sets on the cell segmentation and classification which includes more than 500,000 annotated nuclei in H&E. The CoNIC challenge paper also analysis state-of-the-art nuclei segmentation and classification methods. The authors should add one of the best performing models in their analysis. I would also suggest the authors to include PQ and froc in the metrics to analyse the results as this is commonly used in this domain for comparison. I would also suggest to compare the results with HoVerNet or HoVerNext which are state-of-the-art algorithms for nuclei instance segmentation. The code for these algorithms is publicly available.
Response 1: Thanks for your valuable suggestions.
Regarding the dataset and the best performing model of the CoNIC challenge you mentioned, we carefully reviewed the relevant literature. As described in the CoNIC challenge publication(Graham et al., 2024), the best performing model in the CoNIC challenge is Stardist, which has been included in our analysis. The patches in CoNIC challenge dataset are extracted from the original Lizard dataset, which has already been included in our research and therefore was not added redundantly.
Regarding the addition of PQ and froc indicators you mentioned, we have carefully considered it. Some models may perform well at the pixel level (hence the high F1 score), but the segmentation of instance boundaries is not accurate enough. The PQ indicator can show this, so we added the PQ indicator, which is specifically shown in Figure 2a-e, and added a detailed description and calculation formula in the Metrics section of Methods.
But for the froc metric, we fully understand that the froc curve is widely used in certain detection-based medical image analysis tasks, especially when evaluating the sensitivity and false positive rate of models at different thresholds. However, in our work, the main focus is on cell segmentation, not object detection or localization. Compared with detection tasks, segmentation models are designed to accurately depict individual instances at the pixel level, which makes the application of the froc curve less intuitive and less informative for such task scenarios. In order to more comprehensively evaluate the performance of the model, we supplemented the introduction of the AP-IoU curve, which can reflect the performance of the model under different degrees of strictness of matching conditions (different IoU thresholds), and can more

carefully demonstrate the robustness and segmentation quality of the model. The relevant content is in the second paragraph of Results, Evaluation on four staining types respectively, Figure 2f-i. We believe that this method can more accurately reflect the actual performance of the model in the segmentation task. Finally, we added the HoverNet model pre-trained on the Kumar dataset (the Kumar dataset contains various tissue type which is similar to CellBinDB). In the benchmark, we only added HoverNet to the comparison on the H&E subset and HoverNet performed well. We did not test HoverNet on other staining types of datasets because: 1. HoveNet is specifically designed for pathology images and has not been trained on fluorescent staining datasets; 2. HoveNet is not a multimodal model and is not scalable. The relevant content is in the first paragraph of Results, Evaluation on four staining types respectively, Figure 2d.
We hope this clarification addresses your concerns and improves the overall rigor of our evaluation.

reference

Graham, S. et al. (2024) ‘CoNIC Challenge: Pushing the frontiers of nuclear detection, segmentation, classification and counting’, Medical Image Analysis, 92, p. 103047. Available at: https://doi.org/10.1016/j.media.2023.103047.

Best wishes,
Mei
