# Supplementary material for: CellBinDB: a large-scale multimodal annotated dataset for cell segmentation with benchmarking of universal models
Source: Gigascience. 2025 Jun 24;14:giaf069. doi: 10.1093/gigascience/giaf069 (PMC12206155; doi:10.1093/gigascience/giaf069)
Supplement: giaf069_Reviewer_1_Report_Original_Submission — Jeff Rhoades -- 1/22/2025 [file giaf069_reviewer_1_report_original_submission.pdf]

Reviewer Report

Title: CellBinDB: A Large-Scale Multimodal Annotated Dataset for Cell Segmentation with Benchmarking of Universal Models

Version: Original Submission **Date:** 1/22/2025

Reviewer name: Jeff Rhoades

Reviewer Comments to Author:

General comments:
1. Dataset Innovation: CellBinDB offers a significant improvement over existing datasets with its diversity of staining types (DAPI, ssDNA, H&E, mIF) and broad tissue coverage, including normal and diseased samples.
2. Benchmarking of Models: The evaluation of seven state-of-the-art segmentation algorithms provides valuable insights for researchers selecting tools for various imaging modalities.
3. Analysis of Influencing Factors: The manuscript thoroughly examines biological (e.g., cell morphology) and technical (e.g., image gradient) factors affecting model performance, providing practical recommendations for improving segmentation outcomes.
4. Preprocessing Impact: Demonstrating the effectiveness of preprocessing (e.g., grayscale conversion for H&E images) is an immediately actionable takeaway for practitioners. However, authors should apply preprocessing uniformly to all segmentation approaches, not just those that did poorly initially.
Major Areas for Improvement:
1. Preprocessing Uniformity:
* Apply preprocessing steps uniformly across all segmentation approaches to ensure fair comparisons and avoid bias.
2. Inclusion of Cellpose3 Training Dataset:
* The manuscript should include the dataset used for training Cellpose3 in its comparisons. Cellpose3's superior generalist model performance is emphasized, yet the absence of its training dataset in the comparisons raises questions about robustness of the benchmarking.
3. Evidence of Dataset Utility:
* While the dataset's benchmarking is well-done, the manuscript does not provide evidence that models trained on CellBinDB outperform those trained on other datasets. Addressing this, though potentially out of scope, would strengthen the manuscript's impact.
4. Figure Panels:
* Labeling in figure panels should be clearer to enhance interpretability. For instance, indicate whether the instance or semantic masks are being shown and consider making instance segmentation masks colorful to highlight unique IDs.
* Semantic masks could be omitted if space is constrained, as they are largely redundant with instance masks.
* Ensure figures are spaced more evenly throughout the text, ideally located near their first references, to improve readability.

5. Abstract Clarity:
* The abstract should better reflect the intellectual contributions of the analysis of segmentation performance factors (i.e. cell morphology and image gradients).
6. Normalization Methods:
* Provide details on how cell morphology indicators are normalized in the methods section to ensure reproducibility and clarity.
7. Explanation of Image Gradient:
* The discussion of gradient magnitude and its calculation using the Sobel operator requires more accessible language. Not all readers will be familiar with this concept, so additional context is essential.
8. Tissue Classification:
* Group related tissues, such as "brain," "half brain," and "cerebellum," under a common "neural tissue" category for easier interpretation and analysis.
Additional Suggestions:
* Address grammatical errors and improve clarity in some sections, such as the benchmarking pipeline description.
* Replace vague terms like "ML-based" when referring to CellProfiler with specific algorithmic descriptions.
* Including public datasets, such as Cellpose, to create a unified, all-inclusive CellBinDB dataset might significantly enhance the resource's utility for machine learning practitioners.

Methods

Are the methods appropriate to the aims of the study, are they well described, and are necessary controls included? Choose an item.

Conclusions

Are the conclusions adequately supported by the data shown? Choose an item.

Reporting Standards

Does the manuscript adhere to the journal's guidelines on [minimum standards of reporting?]() Choose an item.

Choose an item.

Statistics

Are you able to assess all statistics in the manuscript, including the appropriateness of statistical tests used? Choose an item.

Quality of Written English

Please indicate the quality of language in the manuscript: Choose an item.

Declaration of Competing Interests

Please complete a declaration of competing interests, considering the following questions:

- Have you in the past five years received reimbursements, fees, funding, or salary from an organisation that may in any way gain or lose financially from the publication of this manuscript, either now or in the future?
- Do you hold any stocks or shares in an organisation that may in any way gain or lose financially from the publication of this manuscript, either now or in the future?
- Do you hold or are you currently applying for any patents relating to the content of the manuscript?
- Have you received reimbursements, fees, funding, or salary from an organization that holds or has applied for patents relating to the content of the manuscript?
- Do you have any other financial competing interests?
- Do you have any non-financial competing interests in relation to this paper?

If you can answer no to all of the above, write 'I declare that I have no competing interests' below. If your reply is yes to any, please give details below.

I declare that I have no competing interests.

I agree to the open peer review policy of the journal. I understand that my name will be included on my report to the authors and, if the manuscript is accepted for publication, my named report including any attachments I upload will be posted on the website along with the authors' responses. I agree for my report to be made available under an Open Access Creative Commons CC-BY license (http://creativecommons.org/licenses/by/4.0/). I understand that any comments which I do not wish to be included in my named report can be included as confidential comments to the editors, which will not be published.

Choose an item.

To further support our reviewers, we have joined with Publons, where you can gain additional credit to further highlight your hard work (see: https://publons.com/journal/530/gigascience). On publication of this paper, your review will be automatically added to Publons, you can then choose whether or not to claim your Publons credit. I understand this statement.

Yes Choose an item.
