# Supplementary material for: CellBinDB: a large-scale multimodal annotated dataset for cell segmentation with benchmarking of universal models
Source: Gigascience. 2025 Jun 24;14:giaf069. doi: 10.1093/gigascience/giaf069 (PMC12206155; doi:10.1093/gigascience/giaf069)
Supplement: giaf069_Reviewer_2_Report_Original_Submission — Shan Raza -- 2/4/2025 [file giaf069_reviewer_2_report_original_submission.pdf]

Reviewer Report

Title: CellBinDB: A Large-Scale Multimodal Annotated Dataset for Cell Segmentation with Benchmarking of Universal Models

Version: Original Submission **Date:** 2/4/2025

Reviewer name: Shan Raza

Reviewer Comments to Author:

The paper presents a multimodal data set for cell segmentation and benchmarking. The major strength of the dataset is its multimodal nature and including both mouse and human tissue. The paper analyses existing data sets and the performance of state-of-the-art methods. However, the authors missed one of the biggest data sets on the cell segmentation and classification which includes more than 500,000 annotated nuclei in H&E https://www.sciencedirect.com/science/article/pii/S1361841523003079. The CoNIC challenge paper also analysis state-of-the-art nuclei segmentation and classification methods. The authors should add one of the best performing models in their analysis. I would also suggest the authors to include PQ and froc in the metrics to analyse the results as this is commonly used in this domain for comparison. I would also suggest to compare the results with HoVerNet or HoVerNext (https://github.com/digitalpathologybern/hover_next_train) which are state-of-the-art algorithms for nuclei instance segmentation. The code for these algorithms is publicly available.

Methods

Are the methods appropriate to the aims of the study, are they well described, and are necessary controls included? Choose an item.

Conclusions

Are the conclusions adequately supported by the data shown? Choose an item.

Reporting Standards

Does the manuscript adhere to the journal's guidelines on [minimum standards of reporting?](#) Choose an item.

Choose an item.

Statistics

Are you able to assess all statistics in the manuscript, including the appropriateness of statistical tests used? Choose an item.

Quality of Written English

Please indicate the quality of language in the manuscript: Choose an item.

Declaration of Competing Interests

Please complete a declaration of competing interests, considering the following questions:

- Have you in the past five years received reimbursements, fees, funding, or salary from an organisation that may in any way gain or lose financially from the publication of this manuscript, either now or in the future?
- Do you hold any stocks or shares in an organisation that may in any way gain or lose financially from the publication of this manuscript, either now or in the future?
- Do you hold or are you currently applying for any patents relating to the content of the manuscript?
- Have you received reimbursements, fees, funding, or salary from an organization that holds or has applied for patents relating to the content of the manuscript?
- Do you have any other financial competing interests?
- Do you have any non-financial competing interests in relation to this paper?

If you can answer no to all of the above, write 'I declare that I have no competing interests' below. If your reply is yes to any, please give details below.

I declare that I have no competing interests.

I agree to the open peer review policy of the journal. I understand that my name will be included on my report to the authors and, if the manuscript is accepted for publication, my named report including any attachments I upload will be posted on the website along with the authors' responses. I agree for my report to be made available under an Open Access Creative Commons CC-BY license (http://creativecommons.org/licenses/by/4.0/). I understand that any comments which I do not wish to be included in my named report can be included as confidential comments to the editors, which will not be published.

Choose an item.

To further support our reviewers, we have joined with Publons, where you can gain additional credit to further highlight your hard work (see: https://publons.com/journal/530/gigascience). On publication of this paper, your review will be automatically added to Publons, you can then choose whether or not to claim your Publons credit. I understand this statement.

Yes Choose an item.
